# Microfluidic Profiling of an Immune Cell‐Associated Extracellular Vesicle Signature for Active Tuberculosis Diagnosis and Treatment Monitoring

**DOI:** 10.1002/jev2.70395

**Published:** 2026-11-03

**Authors:** Eun Ju Lee, Cheng Nie, Sunyoung Park, Yun‐Jeong Kang, Minji Kang, Jake Whang, Go‐Eun Choi, Hyejon Lee, San‐Nae Cho, Kyung‐A Hyun, Sunghyun Kim, Hyo‐Il Jung, Jungho Kim

**Affiliations:** ^1^ Department of Clinical Laboratory Science, College of Health Sciences Catholic University of Pusan Busan Republic of Korea; ^2^ Next‐Generation Industrial Field‐Based Specialist Program for Molecular Diagnostics, Brain Busan 21 Plus Project, Graduate School Catholic University of Pusan Busan Republic of Korea; ^3^ School of Mechanical Engineering Yonsei University Seoul Republic of Korea; ^4^ School of Mechanical and Electrical Engineering Zaozhuang University Zaozhuang Shandong China; ^5^ Department of Biomedical Technology Kangwon National University Chuncheon Republic of Korea; ^6^ Department of Biomedical Laboratory Science Dong‐Eui Institute of Technology Busan Republic of Korea; ^7^ Korea Mycobacterium Resource Center (KMRC), Department of Research and Development The Korean Institute of Tuberculosis Osong Republic of Korea; ^8^ Clinical Vaccine Research Section, International Tuberculosis Research Center Changwon Republic of Korea; ^9^ Institute of Immunology and Immunological Disease Yonsei University College of Medicine Seoul Republic of Korea; ^10^ School of Biopharmaceutical and Medical Sciences Sungshin Women's University Seoul Republic of Korea; ^11^ Basic Science Institute Sungshin Women's University Seoul Republic of Korea; ^12^ The DABOM Inc., 50 Yonsei‐ro Seodaemun‐gu Seoul Republic of Korea; ^13^ The Institute of Biotechnology and Genetic Engineering Chulalongkorn University Bangkok Thailand

**Keywords:** active tuberculosis, biomarker identification, extracellular vesicle, microfluidic chip

## Abstract

Rapid and accurate diagnosis of active tuberculosis (ATB) remains challenging, particularly in paucibacillary cases, where conventional pathogen detection often fails. Plasma extracellular vesicles carrying innate immune cell‐associated surface proteins, termed tuberculosis‐associated immune cell extracellular vesicles (TB‐IEVs), were investigated as biomarkers of ATB and integrated into a multichannel microfluidic platform. Whole‐blood transcriptome profiling of patients with ATB and healthy controls (HCs) enabled the systematic selection of four innate immune cell‐associated surface markers (RAB8A, TYROBP, C3AR1 and LILRA2). Marker validation using a bead‐based immunoprecipitation assay confirmed elevated TB‐IEV levels in ATB plasma. This signature was implemented on an 8‐channel microfluidic chip, enabling parallel fluorescence analysis of only 10 µL of plasma within 30 min. In a clinical cohort (25 patients with ATB and 24 HCs), the TB‐IEV multichannel chip achieved an area under the curve of 0.98. The signature also distinguished ATB from other respiratory diseases (*n* = 40) with an AUC of 0.95 and provided complementary diagnostic information in ATB patients with negative results on individual conventional tests. Longitudinal monitoring showed markedly decreased TB‐IEV levels following anti‐tuberculosis treatment. Collectively, this work demonstrated the potential of TB‐IEV profiling as a rapid, minimally invasive approach for ATB diagnosis and treatment monitoring.

## Graphical abstract

Integrating microfluidic parallel detection with an innate immune extracellular vesicle signature enables rapid active tuberculosis diagnosis from 10 µL of plasma in 30 minutes, effectively discriminating non‐tuberculous respiratory diseases and monitoring treatment response.

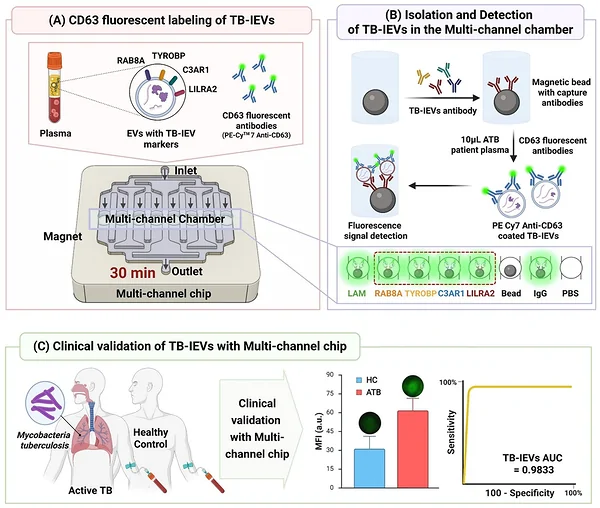

## 1 Introduction

Tuberculosis (TB), caused by *Mycobacterium tuberculosis* (MTB), remains a persistent global health crisis. Despite temporary fluctuations during the COVID‐19 pandemic, diagnostic delays and disruptions in healthcare services threaten to increase the burden of untreated TB (Ravimohan et al. 2018; World Health Organization 2022). Current diagnostic paradigms primarily rely on respiratory specimens, such as sputum smear microscopy, culture and nucleic acid amplification tests (NAATs) (Mann et al. 2024; Solanki et al. 2024; Hamada et al. 2021). However, these methods have intrinsic limitations; culture is time‐consuming, microscopy lacks sensitivity and NAATs are rapid, resource‐intensive and often yield false‐negative results in paucibacillary cases (Musisi et al. 2024; Seki et al. 2018; Liang et al. 2024; Theron et al. 2016; Hou et al. 2022; Dorman et al. 2018). Importantly, the reliance on sputum renders diagnosis particularly challenging in children, older adults and immunocompromised patients who cannot produce adequate samples (Nathavitharana et al. 2022). Consequently, there is an urgent need for rapid, non‐sputum‐based diagnostic modalities capable of detecting active TB (ATB) with high sensitivity, regardless of bacterial burden (Hoang et al. 2021; Heo et al. 2025).

Blood‐based biomarkers are promising alternatives, with extracellular vesicles (EVs) emerging as highly attractive candidates for non‐sputum‐based diagnosis (Mizenko et al. 2024). EVs are nanosized membrane‐bound particles released by host cells that encapsulate molecular signatures reflective of their cell of origin (Samanta et al. 2018; Buzas 2023; Kumar et al. 2024). Their phospholipid bilayers confer high stability in circulation, making them ideal candidates for clinical diagnostics (Groß et al. 2024; Yu et al. 2024; Mir and Goettsch 2020; Hallal et al. 2022; Pegtel and Gould 2019; Arya et al. 2024; Herrmann et al. 2021). In TB pathogenesis, MTB actively manipulates host immune cells—macrophages, dendritic cells and neutrophils—triggering profound immunometabolic reprogramming (Chandra et al. 2022; Sheedy and Divangahi 2021). We hypothesized that this host‐pathogen interaction imprints a specific molecular signature onto the surface of immune cell‐derived EVs, creating a unique “TB‐associated” phenotype (Salgueiro et al. 2024).

Although EV‐based approaches are promising, most existing strategies analyse intravesicular cargo such as MTB‐derived antigens or host RNAs (Zheng et al. 2022). Although these markers provide valuable biological insights, accessing luminal contents typically requires EV lysis and multistep processing, which can hinder the development of rapid point‐of‐care (POC) systems (Shao et al. 2018). In contrast, EV surface proteins are directly accessible for affinity‐based capture, enabling a streamlined “capture‐and‐detect” workflow without sample pretreatment. Nevertheless, the specific surface protein repertoire that distinguishes ATB‐derived EVs from those of healthy individuals or patients with latent infections remains poorly defined, representing a critical gap in the field.

To bridge this gap, we identified a distinct subset of EVs, termed TB‐associated immune cell‐extracellular vesicles (TB‐IEVs), which are characterized by surface proteins associated with innate immune responses in active disease. Using comprehensive transcriptome profiling of whole blood from patients with ATB and healthy controls (HCs), we systematically filtered immune cell‐associated, membrane‐localized markers to identify RAB8A, TYROBP, C3AR1 and LILRA2 protein levels as a robust four‐marker signature (Seto et al. 2011; Zhang et al. 2024).

Translating these biomarkers into clinically deployable tools requires a platform that balances sensitivity and speed. Conventional EV isolation methods (e.g., ultracentrifugation) are labour‐intensive and poorly suited for clinical routines. To overcome these barriers, we engineered a custom multichannel microfluidic chip for parallel immunocapture and fluorescence detection. This platform enables the automated analysis of TB‐IEVs from only 10 µL of plasma within 30 min, substantially enhancing diagnostic throughput and accessibility (Figure 1).

**FIGURE 1:**
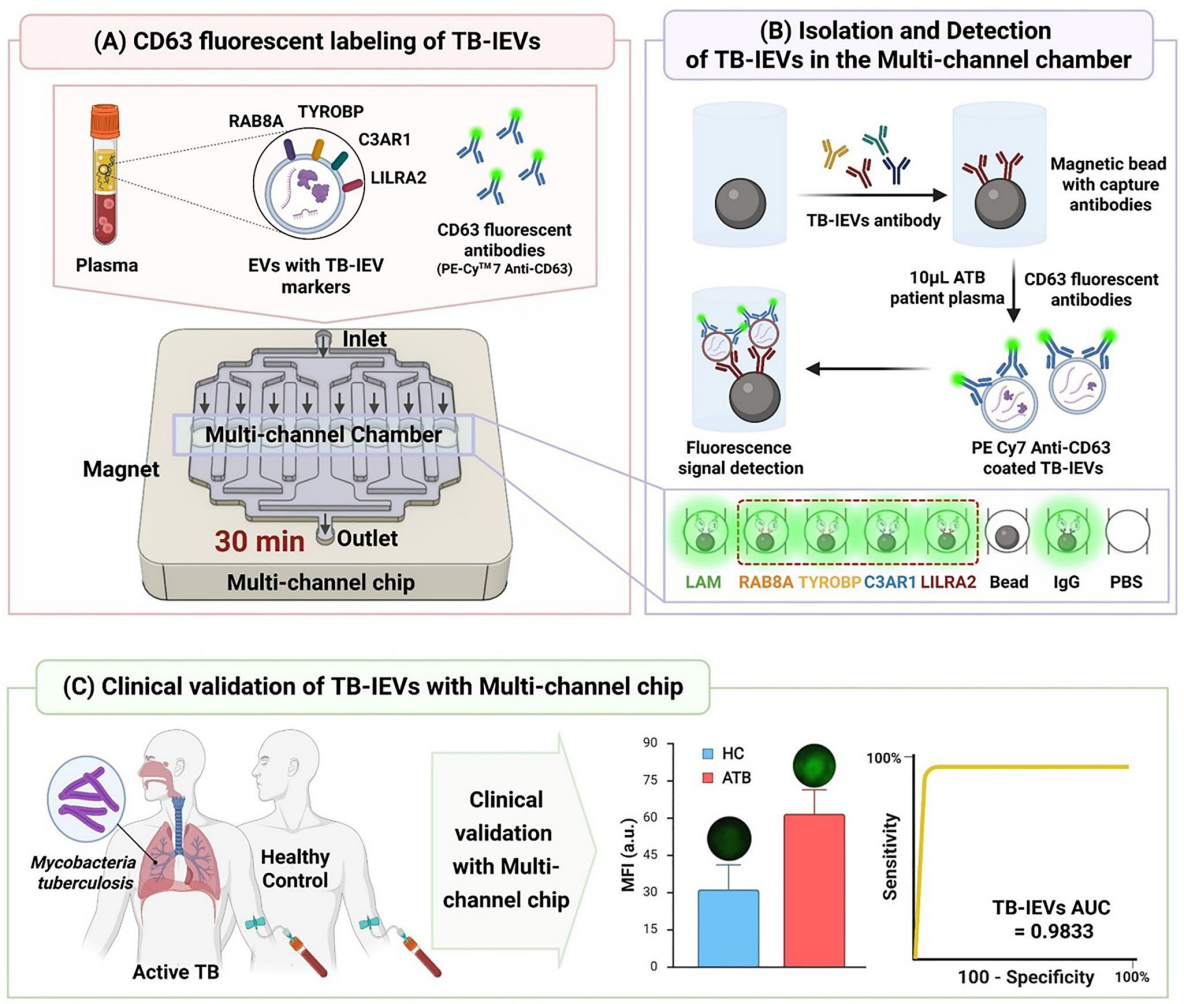
Schematic illustration of establishment and clinical validation of a multichannel chip‐based tuberculosis diagnosis platform for detecting TB‐IEVs. Created with BioRender.com. Kim, J. (2026) https://BioRender.com/inc1wc9. (A) Schematic overview of multichannel chip analysis of TB‐IEVs. The 10 µL volumes of plasma samples, containing EVs with TB‐IEV markers (RAB8A, TYROBP, C3AR1 and LILRA2), are initially incubated with PE‐Cy7 conjugated anti‐CD63 antibodies. These fluorescently labelled samples are loaded into the inlet of the multichannel chip. Within the 30 min incubation period, fluorescence signals of TB‐IEVs are detected. (B) Isolation and detection of TB‐IEVs in the multichannel chamber. Immunomagnetic beads, coupled with target antibodies, captured fluorescently labelled TB‐IEVs, enabling the rapid fluorescence signal acquisition. Anti‐IgG magnetic beads serve as positive controls, and nature magnetic beads and PBS are used as negative controls. (C) Clinical validation of TB‐IEVs using multichannel chip in the plasma from patients with active tuberculosis and healthy controls. ATB, active tuberculosis; AUC, area under the curve; EVs, extracellular vesicles; MFI, median fluorescence Intensity; PBS, phosphate‐buffered saline; ROC, receiver operating characteristic curve; TB‐IEVs, tuberculosis‐associated immune cell extracellular vesicles.

In this study, we demonstrated that circulating TB‐IEVs carrying the four innate immune cell‐associated markers were elevated in patients with ATB and could be detected using a multichannel microfluidic platform. The TB‐IEV signature discriminated ATB from both HCs and patients with other respiratory diseases and provided complementary diagnostic information in patients with established ATB who had negative results on individual conventional diagnostic tests. Longitudinal analysis further demonstrated changes in TB‐IEV levels following anti‐tuberculosis treatment. By integrating systems‐level biomarker discovery with microfluidic engineering, this study presents a rapid, low‐volume, plasma‐based approach with potential utility for ATB diagnosis and treatment monitoring.

## 2 Results

### 2.1 Transcriptomic Discovery of Immune‐Specific Surface Markers on TB‐IEVs

To identify robust TB‐IEV surface markers, we analysed whole blood transcriptomes from 12 patients with ATB and 14 HCs. A systematic multistep screening strategy (Figure 2A) revealed distinct molecular signatures differentiating ATB from HCs (*p* < 0.01; Figure S1). Of the 1064 differentially expressed genes, 297 were upregulated (Figure 2B; Table S1). To select immune cell‐associated markers with surface accessibility for plasma‐based EV detection, we applied stringent filtering criteria. Genes highly expressed in immune cells (Human Protein Atlas) and encoding transmembrane domains suitable for antibody capture (UniProt) were prioritized, resulting in a shortlist of 18 candidates, from which RAB8A, TYROBP, C3AR1 and LILRA2 were selected as the four TB‐IEV surface markers (Figure 2C,D).

**FIGURE 2:**
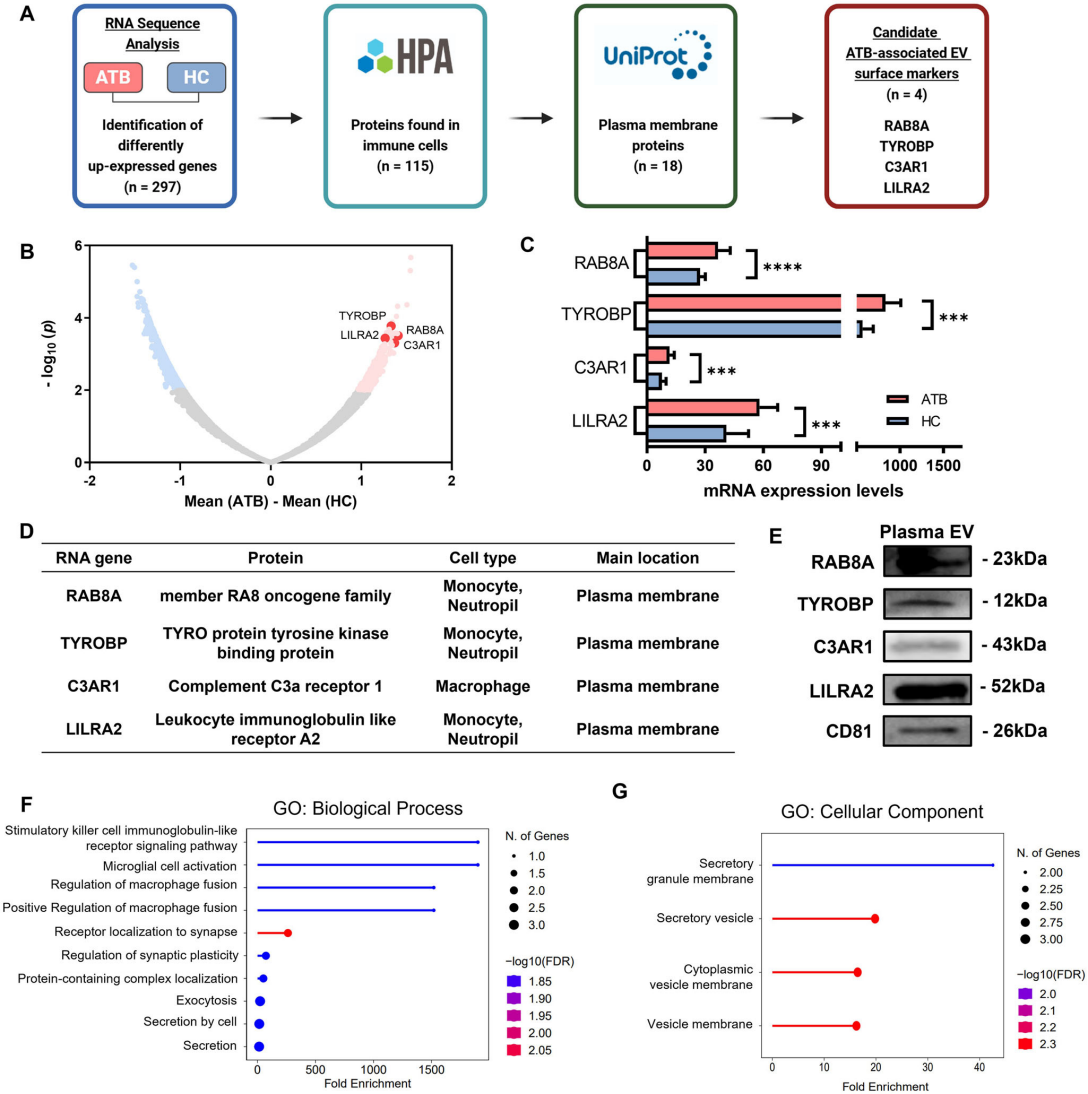
Identification and validation of TB‐IEV markers based on transcriptomic profiles. (A) Schematic workflow illustrating the identification of potential markers of TB‐IEV. (B) Volcano plot showing differentially expressed genes between HCs and patients with ATB. A total of 1064 genes were significantly differentially expressed, including 297 upregulated and 767 downregulated genes (*p* < 0.01). (C) Expression levels of the four selected TB‐IEV markers in whole blood samples of HCs and patients with ATB. (D) Summary of the characteristics of the four selected TB‐IEV markers. (E) Western blot analysis of plasma‐derived total EVs from patients with ATB confirming the presence of TB‐IEV markers. CD81 serves as an EV marker control. (F, G) Gene Set Enrichment Analysis of Gene Ontology (GO) terms: (F) biological processes and (G) cellular components for the four selected TB‐IEV markers. ATB, active tuberculosis; EVs, extracellular vesicles; FDR, false discovery rate; HCs, healthy controls; TB‐IEVs, tuberculosis‐associated immune cell extracellular vesicles. ****p < 0.0001, ***p < 0.001.

We validated the presence of these markers in circulating plasma EVs. Nanoparticle tracking analysis (NTA) and transmission electron microscopy (TEM) confirmed that plasma‐derived EVs exhibited a typical size distribution (50–200 nm; mean, 116 nm) and morphology (Figure S2A,B). Western blot analysis confirmed the expression of all four selected markers on plasma EVs, along with the classic EV marker CD81 (Figure 2E). Gene ontology (GO) analysis further supported the biological relevance of these markers. Biological processes were enriched in the immune activation pathways, including microglial activation and macrophage fusion (Figure 2F). Cellular component analysis revealed notable enrichment in secretory vesicles and granule membranes (Figure 2G), supporting their suitability as EV biomarkers of immune origin.

### 2.2 Validation of TB‐IEV Markers Using a Bead‐Based Assay

To capture TB‐IEVs from plasma, we established an immunomagnetic bead‐based assay targeting the four selected markers, as illustrated in the schematic workflow (Figure 3A). Flow cytometry confirmed successful antibody conjugation and complex stability for up to 15 days (Figure S3). Prior to clinical evaluation, we characterized the marker‐captured EVs using multiple complementary modalities. Scanning electron microscopy (SEM) imaging revealed spherical vesicles bound to the bead surface (Figure 3B). Confocal microscopy and western blotting further demonstrated the presence of the canonical EV marker CD63 in the marker‐captured preparations, supporting their EV‐associated nature (Figure 3C,D). To further characterize their physical properties, captured EVs were eluted from the beads. TEM imaging of the released vesicles revealed intact bilayer structures (Figure 3E) and NTA quantitatively verified a typical EV size distribution (Figure 3F).

**FIGURE 3:**
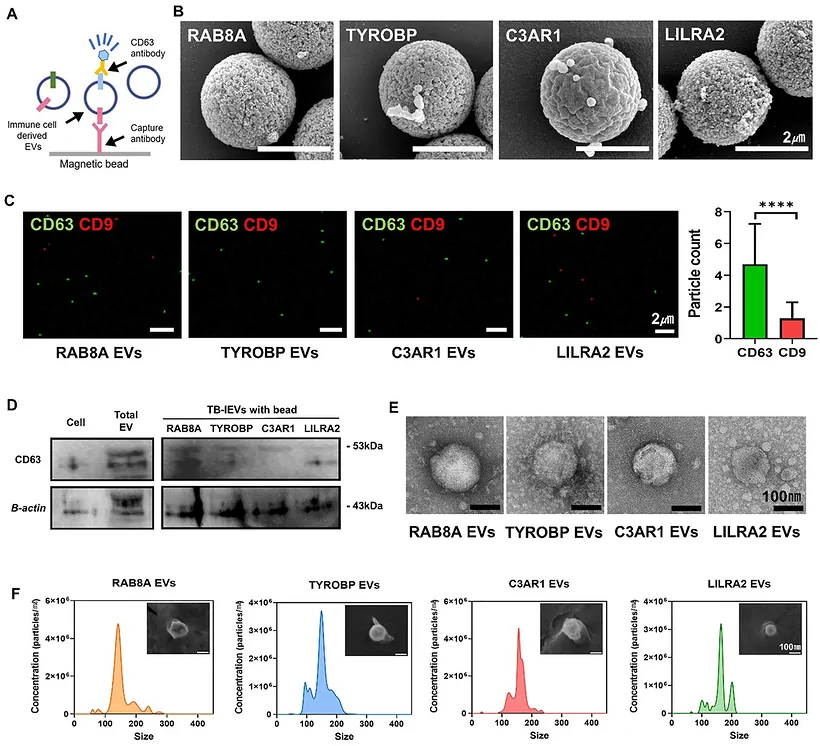
Characterization of target‐captured TB‐IEVs. (A) Schematic illustration of the immunoprecipitation method using antibody coupled magnetic beads for TB‐IEVs detection. (B) Scanning electron microscopy (SEM) images of TB‐IEVs captured by immunomagnetic beads (scale bar, 2 µm). (C) Fluorescence validation of captured TB‐IEVs using confocal microscopy, showing colocalization of CD63 labelled with PE‐Cy7 (green) and CD9 labelled with PerCp (red) (scale bar, 2 µm). Quantification shows significantly higher detection of TB‐IEVs using CD63 than using CD9. (D) Western blot analysis of immunoprecipitated TB‐IEVs confirming successful EV enrichment. (E) Transmission electron microscopy images (scale bar, 100 nm) and (F) nanoparticle tracking analysis with corresponding SEM images (scale bar, 100 nm) of TB‐IEVs in the plasma for validating the size distribution and morphology. EVs, extracellular vesicles; TB‐IEVs, tuberculosis‐associated immune cell extracellular vesicles. ****p < 0.0001.

We evaluated the diagnostic performance of TB‐IEVs in a pilot cohort (10 HCs and 24 patients with ATB). Among the conventional EV markers, CD63 and CD81 failed to distinguish ATB from HCs (*p* > 0.05), whereas CD9 showed a significant difference (*p* < 0.05) but showed only moderate diagnostic accuracy (area under the curve [AUC] = 0.76) (Figure 4A,B). In contrast, the four TB‐IEV markers showed greater discriminatory performance than the conventional EV markers in this cohort. As shown in Figure 4C, all four markers were significantly elevated in patients with ATB, with TYROBP and C3AR1 showing the largest differences between the groups (*p* < 0.0001). Receiver operating characteristic (ROC) curve analysis showed AUCs of 0.96 and 0.95 for TYROBP and C3AR1, respectively (Figure 4D; Table S2). The combined four‐marker signature achieved an AUC of 0.99 in this pilot cohort (Figure 4D). These distinct expression patterns were further visualized using a heat map, which showed separation between patients with ATB and HCs based on TB‐IEV intensity, whereas conventional markers showed overlapping profiles (Figure 4E). Collectively, the combinatorial signature demonstrated a sensitivity of 95.8% and a specificity of 90.0%, for distinguishing ATB from HCs in this initial validation cohort (Figure S4).

**FIGURE 4:**
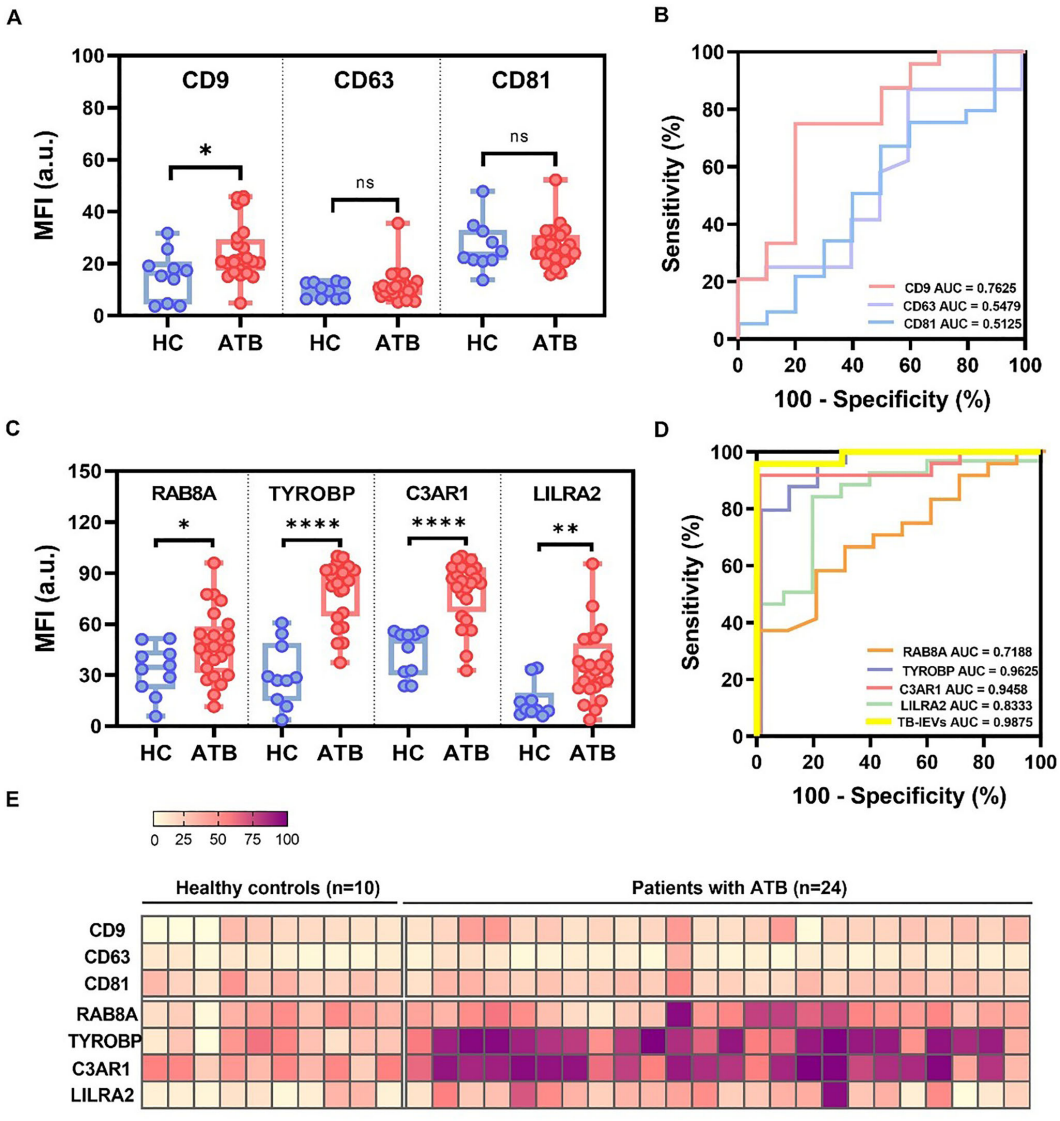
Diagnostic performance of TB‐IEV markers in the pilot validation cohort. (A) Expression levels and (B) ROC curve analysis of EVs captured using immunomagnetic beads conjugated with canonical EV markers (CD9, CD63 and CD81) from plasma samples of HCs and patients with ATB. (C) Expression levels and (D) ROC curve analysis of EVs captured using immunomagnetic beads conjugated with antibodies against the four TB‐IEV markers (RAB8A, TYROBP, C3AR1 and LILRA2) in plasma samples from HCs and patients with ATB. (E) Heat map comparing fluorescence intensity profiles of canonical EV markers and TB‐IEV markers in HCs and patients with ATB. ATB, active tuberculosis; AUC, area under the curve; EVs, extracellular vesicles; HCs, healthy controls; MFI, median fluorescence intensity; ROC, receiver operating characteristic; TB‐IEVs, tuberculosis‐associated immune cell extracellular vesicles. ****p < 0.0001, **p < 0.01 and *p < 0.05. ns, not significant.

### 2.3 Engineering a Multichannel Microfluidic Chip for Rapid, High‐Sensitivity Detection

To translate these biomarkers to a rapid POC platform, we developed an 8‐channel microfluidic chip. Computational fluid dynamics simulations confirmed uniform flow distribution across all channels, ensuring reliable parallel processing without stagnation (Figure S5). Magnetic field simulations showed consistent peak intensities ranging from 0.099 to 0.121T along the a‐a’ section of the channel. This magnetic distribution generated a strong magnetic force (>1.7 × 10^−^^9^ N) exceeding the critical retention threshold (Figure S6A–C). Crucially, the sharp decrease in magnetic flux density between adjacent chambers confirmed that the inter‐chamber spacing was sufficient to prevent magnetic crosstalk, ensuring independent and stable bead capture in each chamber (Figure S6C). Experimental validation demonstrated >90% bead capture efficiency within 15 s across all chambers (Figure S6D).

We then compared the detection performance of the multi‐channel chip against a conventional microcentrifuge tube assay. The chip workflow (Figure 5A) involves sequential steps of sample injection, binding and washing, utilizing preloaded antibody‐functionalized bead and manual injection for simplified operation. This design leverages spatial confinement and continuous flow to enhance the local encounter probability between EVs and beads, effectively concentrating targets within the reaction chamber. As shown in Figure 5B,C, the chip consistently generated significantly stronger fluorescence signals than the tube method across a wide concentration range (10^4^–10^7^ EVs/mL). Quantitative analysis further highlighted the superior detection capability of the multi‐channel chip, particularly at the lower concentration limit (Figure 5D). In the micro centrifuge tube assay, fluorescence signals at 10^4^ EVs/mL were nearly indistinguishable from blank levels, especially for LAM (a mycobacterial antigen), C3AR1 and LILRA2 markers, making reliable detection challenging. In contrast, the multi‐channel chip produced signals that were clearly distinct from the blank across all tested biomarkers even at 10^4^ EVs/mL, yielding a markedly improved signal‐to background ratio. This represents a significant enhancement in signal‐to‐noise ratio, enabling the detection of trace levels of EVs that are typically undetectable in bulk assays. Notably, TB‐IEV markers (RAB8A, TYROBP, C3AR1 and LILRA2) exhibited much stronger signal intensities than LAM, highlighting the superiority of host‐derived EV markers for high‐sensitivity detection.

**FIGURE 5:**
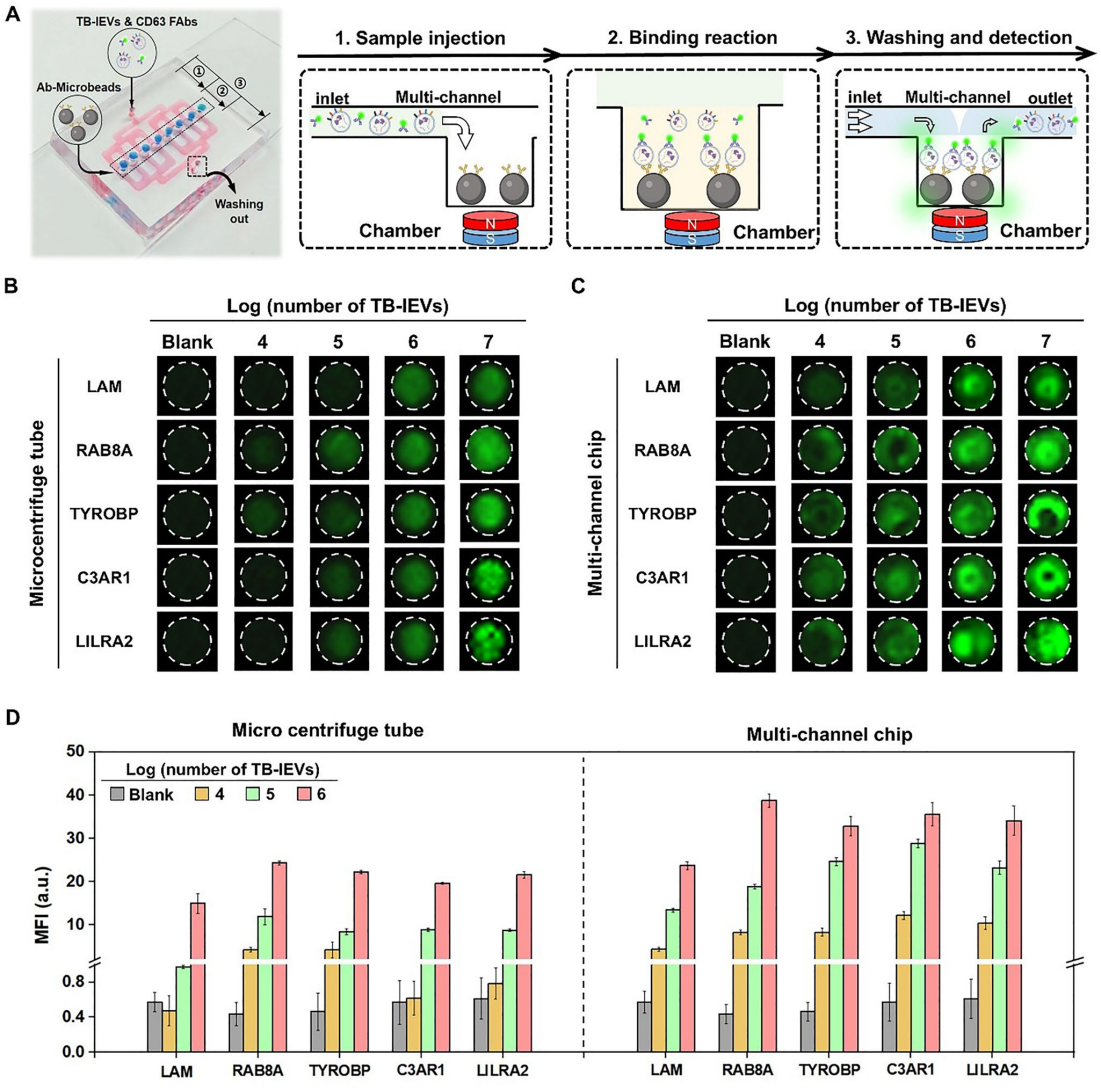
Comparison of TB‐IEV detection efficiency between conventional microcentrifuge tube assay and the multichannel microfluidic chip. (A) Schematic workflow of TB‐IEV detection using the multichannel microfluidic chip. TB‐IEVs labelled with anti‐CD63 fluorescent antibodies are introduced into the chip, followed by binding to antibody‐conjugated magnetic beads, magnetic enrichment within individual chambers, washing and fluorescence signal acquisition. (B) Fluorescence images of TB‐IEVs captured using antibody‐coated magnetic beads in a conventional microcentrifuge tube across increasing input concentrations (log TB‐IEVs = 4–7). (C) Fluorescence images of TB‐IEVs detected using the multichannel chip under identical TB‐IEV input concentrations. The multichannel chip exhibits stronger and more uniform fluorescence signals than the tube‐based method. (D) Quantification of median fluorescence intensity (MFI) for TB‐IEV markers (RAB8A, TYROBP, C3AR1, LILRA2) and the mycobacterial antigen LAM using the tube‐based assay. Increasing TB‐IEV concentrations yield incremental fluorescence responses. Quantification of MFI for each TB‐IEV marker obtained from the multichannel chip. The microfluidic platform demonstrates markedly enhanced signal strength and dynamic range compared with the tube‐based approach. TB‐IEVs, tuberculosis‐associated immune cell extracellular vesicles.

### 2.4 Diagnostic Performance of the Multichannel Chip Compared With ELISA and Conventional Diagnostic Methods

We compared the diagnostic performance of the multichannel chip with that of a commercial ELISA using paired plasma samples from 25 patients with ATB and 24 HCs. Although ELISA detected elevated signals for TB‐IEV markers (Figure 6A), its diagnostic performance varied among the individual markers (AUC 0.57–0.91; Figure 6B). In contrast, the multichannel chip showed improved discriminatory performance. The combined TB‐IEV signature on the chip achieved an AUC of 0.98 for distinguishing ATB from HCs, compared with an AUC of 0.91 for the combined signature measured by ELISA (Figure 6C,D; Table 1). To further assess the discriminatory performance of the signature in a non‐TB disease comparator cohort, we extended the chip analysis to an independent comparator cohort of 40 patients with other respiratory diseases (ORDs), most commonly pneumonia, bronchitis and chronic obstructive pulmonary disease, among other diverse conditions (Table S3). The TB‐IEV marker signals were higher in patients with ATB than in those with ORDs (Figure 6C), and the combined TB‐IEV signature discriminated ATB from ORDs with an AUC of 0.95 (Figure 6E; Table S4 and S5). These findings indicate that the discriminatory performance of the TB‐IEV signature was retained when ATB was compared with a heterogeneous non‐TB respiratory disease cohort, rather than being limited to the comparison with HCs.

**FIGURE 6:**
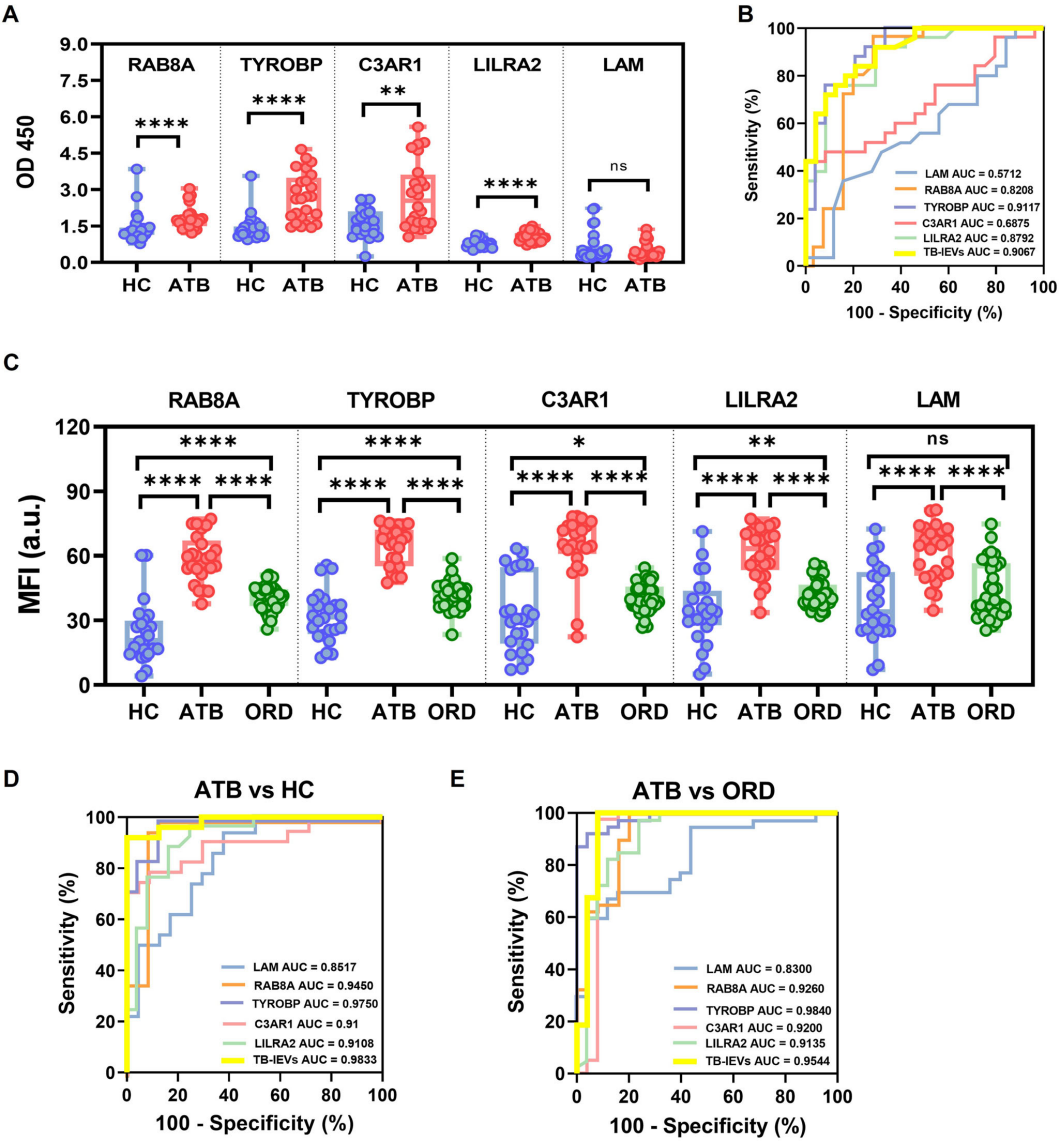
Diagnostic performance of the TB‐IEV multichannel chip compared with ELISA. (A) Signal levels of TB‐IEV markers measured by ELISA in plasma from HCs and patients with ATB. (B) Corresponding ROC curves and AUC values for ELISA‐based detection. (C) Fluorescence intensities of TB‐IEVs detected by the multichannel chip in the plasma samples from HCs, patients with ATB, and patients with ORDs. (D) ROC curves and AUC values for chip‐based discrimination between ATB and HCs. (E) ROC curves and AUC values for chip‐based discrimination between ATB and ORDs. ATB, active tuberculosis; AUC, area under the curve; HCs, healthy controls; MFI, median fluorescence intensity; ORDs, other respiratory diseases; ROC, receiver operating characteristic; TB‐IEVs, tuberculosis‐associated immune cell extracellular vesicles. ****p < 0.0001, **p < 0.01 and *p < 0.05. ns, not significant.

**TABLE 1:** Diagnostic performance of the TB‐IEV multichannel microfluidic chip and commercial ELISA for discriminating active tuberculosis from healthy controls.

Markers		EV levels, median (a.u.)		AUC (95% CI)	Cutoff	Sensitivity	Specificity	*p* value
		HC (*n* = 24)	ATB (*n* = 25)					
Commercial ELISA	LAM	0.34	0.30	0.57 (0.41–0.73)	0.33	52.0%	56.0%	ns
	RAB8A	1.24	1.75	0.82 (0.69–0.95)	1.47	80.0%	79.2%	<0.0001
	TYROBP	1.29	2.62	0.91 (0.83–0.99)	1.59	80.0%	83.3%	<0.0001
	C3AR1	1.58	2.54	0.69 (0.54–0.84)	1.74	60.0%	62.5%	0.0028
	LILRA2	0.74	1.04	0.88 (0.78–0.97)	0.89	76.0%	75.0%	<0.0001
TB‐IEV markers combination		1.31	2.14	0.91 (0.83–0.98)	1.59	80.0%	83.3%	<0.0001
Multi ‐channel microfluidic chip	LAM	33.96	63.01	0.85 (0.74–0.96)	50.72	76.0%	75.0%	<0.0001
	RAB8A	20.55	58.74	0.95 (0.87–1.00)	43.60	92.0%	91.7%	<0.0001
	TYROBP	31.64	67.19	0.98 (0.94–1.00)	51.02	88.0%	87.5%	<0.0001
	C3AR1	30.70	65.29	0.91 (0.83–0.99)	55.43	80.0%	79.2%	<0.0001
	LILRA2	33.52	63.35	0.91 (0.83–0.99)	50.40	84.0%	83.3%	<0.0001
TB‐IEV markers combination		30.23	60.51	0.98 (0.96–1.00)	44.72	92.0%	91.7%	<0.0001

Abbreviations: C3AR1, complement C3a receptor 1; CI, confidence interval; ELISA, enzyme‐linked immunosorbent assay; LILRA2, leukocyte immunoglobulin‐like receptor A2; ns, not significant; RAB8A, Ras‐related protein Rab‐8A; TB‐IEV, tuberculosis‐associated immune cell extracellular vesicle; TYROBP, TYRO protein tyrosine kinase‐binding protein.

We next compared the performance of the TB‐IEV signature with conventional diagnostic tests in patients with established ATB (Figure 7). Conventional tests showed variable positivity rates, including 60.0% (15/25) for MTB PCR, 76.0% (19/25) for chest radiography, 20.0% (5/25) for AFB smear and 32.0% (8/25) and 56.0% (14/25) for AFB agar and broth culture, respectively (Figure 7A,B). In contrast, TB‐IEV positivity was 80.0% (20/25) with the commercial ELISA and 96.0% (24/25) with the multichannel microfluidic chip (Figure 7B,C). To further examine its complementary diagnostic value, we evaluated the TB‐IEV signature in subgroups of patients with established ATB who had negative results on individual conventional diagnostic tests. Importantly, these patients were not necessarily negative across all conventional diagnostic modalities simultaneously, and their diagnosis of ATB had been established independently of the TB‐IEV assay based on microbiological, pathological and/or clinical‐radiological criteria. Among patients who were MTB PCR‐negative (Figure 7D), chest X‐ray‐negative (Figure 7E), or culture‐negative (Figure 7F), the TB‐IEV signature was positive in 10 of 10 MTB PCR‐negative, 6 of 6 chest X‐ray‐negative, and 11 of 11 AFB broth culture–negative patients. Patient‐level results for MTB PCR, AFB staining, AFB culture and chest X‐ray evaluation are provided in Table S6. These findings suggest that TB‐IEV profiling may provide complementary diagnostic information in patients with established ATB when individual conventional diagnostic tests yield negative results. Finally, we evaluated the potential utility of TB‐IEVs for treatment monitoring. Longitudinal analysis of paired plasma samples from eight patients with ATB, collected before initiation of anti‐tuberculosis treatment (T0) and after completion of 6 months of standard therapy (T6), revealed a substantial reduction in TB‐IEV signals following treatment (Figure 7G–K). At T6, all eight patients were negative for MTB PCR, AFB staining, AFB culture and chest X‐ray evaluation showed no radiological evidence of active TB. Consistent with these microbiological and radiological findings, fluorescence intensities of RAB8A, TYROBP, C3AR1 and LILRA2 were markedly reduced at T6 compared with their corresponding pretreatment levels. These findings demonstrate an association between changes in circulating TB‐IEV signals and treatment response and support further investigation of the TB‐IEV signature as a potential biomarker for therapeutic monitoring.

**FIGURE 7:**
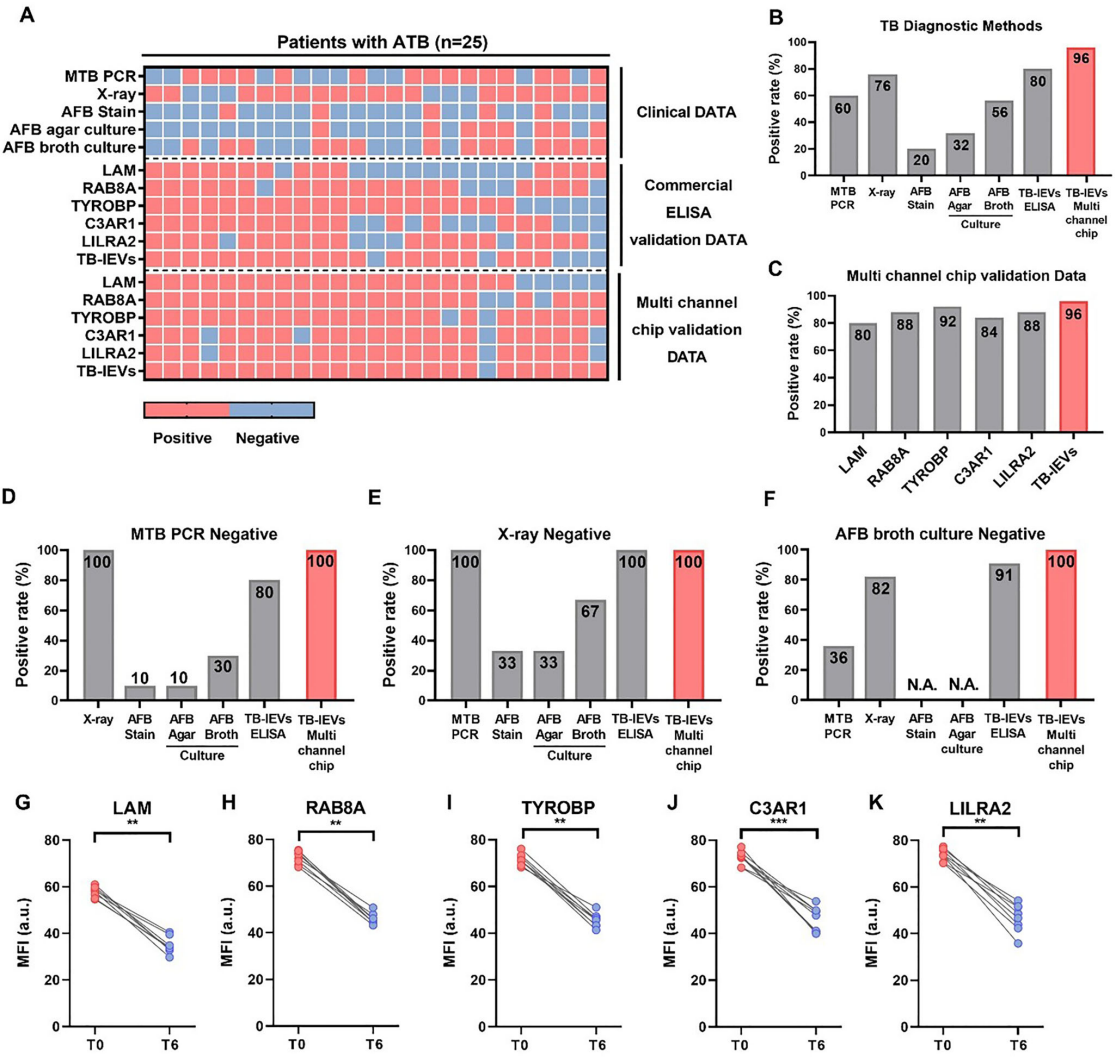
Comparison of TB‐IEV detection using the multichannel microfluidic chip with conventional diagnostic tests and treatment‐associated changes in TB‐IEV signals. (A) Heatmap summarizing test results for MTB PCR, chest X‐ray, AFB smear, AFB agar culture and AFB broth culture, alongside ELISA‐based and multichannel chip‐based TB‐IEV detection in plasma from patients with active TB (*n* = 25). LAM and individual TB‐IEV markers (RAB8A, TYROBP, C3AR1, LILRA2) and their combined signals are shown for ELISA and chip validation datasets. (B) Positive detection rates for conventional diagnostic tests and TB‐IEV detection methods. (C) Positive detection rates for individual TB‐IEV markers and combined TB‐IEVs using the multichannel chip. (D) TB‐IEV and conventional diagnostic test results among patients with established ATB who were negative by MTB PCR. (E) TB‐IEV and conventional diagnostic test results among patients with established ATB who had a negative chest X‐ray evaluation. (F) TB‐IEV and conventional diagnostic test results among patients with established ATB who were negative by AFB broth culture. (G–K) Longitudinal analysis of TB‐IEV marker signals in paired plasma samples collected from patients with ATB before treatment (T0) and after completion of anti‐tuberculosis therapy (T6). Quantitative fluorescence intensity of (G) LAM, (H) RAB8A, (I) TYROBP, (J) C3AR1 and (K) LILRA2 were compared between T0 and T6 to evaluate treatment‐associated changes in TB‐IEV signals. ATB, active tuberculosis; MTB, *Mycobacterium tuberculosis*; TB‐IEVs, tuberculosis‐associated immune cell extracellular vesicles. **p < 0.01, ***p < 0.001, Wilcoxon matched‐pairs signed‐rank test.

## 3 Materials and Methods

### 3.1 Study Population and Setting

For transcriptomic screening of candidate TB‐IEV surface markers, whole‐blood samples were collected from patients attending Yonsei University Severance Hospital, a tertiary referral centre in Seoul, Republic of Korea, between January 2016 and September 2019. A total of 26 participants, comprising 12 patients with active tuberculosis (ATB) and 14 healthy controls (HCs), were included in the transcriptomic discovery cohort. The study was approved by the Institutional Ethics Committee of Severance Hospital (IRB No. 4‐2014‐1108), and written informed consent was obtained from all participants.

Throughout the study, patients with ATB were diagnosed independently of the TB‐IEV assay. In accordance with World Health Organization criteria, bacteriologically confirmed ATB was defined by a positive *Mycobacterium tuberculosis* (MTB) culture from respiratory specimens, a positive MTB PCR result from respiratory specimens, or the presence of caseating granulomas in lung tissue. Patients without bacteriological confirmation were classified as clinically diagnosed ATB when clinical and radiological findings compatible with active TB were accompanied by a favourable clinical and radiological response to anti‐tuberculosis treatment.

HCs had no history of ATB and showed no clinical symptoms suggestive of active TB. They were required to have negative results on the QuantiFERON‐TB Gold In‐Tube Test (QFT‐GIT; QIAGEN, Hilden, Germany) and no radiographic evidence of active TB. Individuals with acute or chronic illnesses that could affect systemic immune responses were excluded from the HC group (Kim et al. 2018; Park et al. 2026).

For clinical validation of the selected TB‐IEV biomarkers and evaluation of the multichannel microfluidic chip, plasma samples were obtained from patients and HCs at Good Samsun Hospital, the Catholic University of Pusan and the National Biobank of Korea, Republic of Korea. Of these samples, 24 patients with ATB and 10 HCs were used for the initial bead‐based biomarker validation, whereas an independent set of 25 patients with ATB and 24 HCs was used for clinical evaluation of the multichannel microfluidic chip. All plasma samples from patients with ATB used for diagnostic and biomarker analyses were collected before the initiation of anti‐tuberculosis treatment. Among the 25 patients with ATB evaluated using the multichannel microfluidic chip, 18 (72.0%) had bacteriologically confirmed ATB and 7 (28.0%) were clinically diagnosed; the diagnostic basis for each patient is provided in Table S6.

To further evaluate the clinical specificity of the TB‐IEV signature, an additional comparator cohort of 40 patients with other respiratory diseases (ORDs) was included. Patients were classified according to their clinically dominant respiratory diagnosis as pneumonia (*n* = 21), bronchitis or acute bronchitis (*n* = 5), chronic obstructive pulmonary disease (COPD; *n* = 2), acute respiratory distress syndrome (*n* = 2), lung carcinoma (*n* = 2), upper respiratory tract infection (*n* = 2), pulmonary embolism (*n* = 2), idiopathic pulmonary fibrosis (*n* = 1), acute interstitial pneumonia (*n* = 1), bronchiectasis (*n* = 1) and empyema (*n* = 1). Concurrent diagnoses and presenting symptoms, where present, are listed in full in Table S3. Active TB was excluded in all ORD patients on clinical, microbiological and radiological grounds and by clinical course (Tables S3 and S5). Ethical approval was obtained from the Institutional Review Board of the Catholic University of Pusan (CUP IRB‐2019‐01‐010, CUP IRB‐2022‐017 and CUP IRB‐2026‐036‐02‐00) and the Public Institutional Review Board appointed by the Ministry of Health and Welfare (Approval No. P01‐202303‐02‐005).

For longitudinal evaluation of treatment response, paired plasma samples were obtained from eight patients with ATB before initiation of anti‐tuberculosis therapy (T0) and after completion of 6 months of standard treatment (T6). At T6, all eight patients were negative for MTB PCR, AFB staining, AFB agar culture and AFB broth culture. Chest X‐ray evaluation also showed no radiological evidence of active TB.

### 3.2 Sample Collection

For RNA sequencing analysis, 3 mL of whole blood was collected in a PAXgene Blood RNA tube (QIAGEN) and stored at −80°C until use.

To clinically validate TB‐IEVs as diagnostic biomarkers using ELISA and the multichannel biochip, blood samples were collected in 10 mL EDTA tubes. Plasma was separated by centrifugation for 10 min at 3000 × *g* and stored at −80°C until use.

### 3.3 Total RNA Isolation

Total RNA was isolated from whole blood samples using a PAXgene Blood miRNA kit (QIAGEN) according to the manufacturer's instructions. The purity and concentration of the isolated total RNA were determined by measuring the absorbance ratio at 260 and 280 nm using a NanoDrop spectrophotometer (Thermo Fisher Scientific, Waltham, MA, USA). The RNA integrity number (RIN) was determined using a BioAnalyzer 2100 (Agilent, Santa Clara, CA, USA), and only samples with RIN ≥ 7.0 were used for RNA sequencing. The isolated total RNA samples were stored at −80°C until use.

### 3.4 RNA Sequencing Analysis

RNA libraries were prepared using the TruSeq Stranded Total RNA Ribo‐Zero Globin Prep Kit (Illumina, San Diego, CA, USA) according to the manufacturer's instructions. An RNA sequencing library was generated on the Illumina HiSeq 2500 platform via cDNA amplification, end‐repair, adenylation of 3´ ends, adapter ligation and amplification. mRNA obtained via sequencing was analysed using MultiExperiment Viewer (MeV 4.9; Perl Foundation, Holland, MI, USA). Heat map and volcano plot analyses were performed to identify differentially expressed mRNAs (cutoff point for a *p* value <0.01).

Based on mRNA sequencing data, we identified ATB‐high expressing mRNA and selected immune cell‐associated candidate biomarkers from the Human Protein Atlas (https://www.proteinatlas.org). Further bioinformatics investigations were performed on UniProt to sort plasma membrane‐located proteins (https://www.uniprot.org).

### 3.5 Total EV Isolation

To determine the presence of selected markers, total EVs were isolated from 100 µL of plasma using a Total Exosome Isolation kit (Invitrogen, Pleasanton, CA, USA) according to the manufacturer's recommendation. First, the plasma sample was centrifuged twice at 2000 × *g* and 1000 × *g* for 20 min to remove cells and debris. Then, 100 µL of the supernatant was transferred to a new microcentrifuge tube, and 50 µL 1 × phosphate‐buffered saline (PBS) was added. After vortexing the mixture, 30 µL of the Exosome Precipitation Reagent was dispensed, and the mixture was inverted until the solution was homogeneous, followed by incubation at room temperature for 10 min. The sample was then centrifuged for 5 min at 10,000 × *g*. The supernatant was aspirated and discarded, and the pellet was resuspended in 1 × PBS. EVs were stored at –20°C until use. Precipitation‐based EV isolation method could co‐isolate many non‐EV components. Accordingly, nanoparticle tracking analysis (NTA) and transmission electron microscopy (TEM) were performed and showed particle size distributions and vesicle‐like morphology consistent with EVs.

### 3.6 EV Capture Using a Magnetic Bead‐Based Immunoprecipitation Assay

To capture TB‐IEVs, magnetic beads (Dynabeads M‐270 Epoxy; Thermo Fisher Scientific) were coupled with antibodies against general EV‐associated markers (CD9, CD63 and CD81) and TB‐IEV markers (RAB8A, TYROBP, C3AR1 and LILRA2) (Table S7). CD9, CD63 and CD81 are members of the tetraspanin family that are commonly enriched in EVs and are widely used as EV‐associated markers for EV detection and characterization. In this study, these tetraspanins were used as general EV‐associated markers, whereas RAB8A, TYROBP, C3AR1 and LILRA2 were evaluated as candidate TB‐IEV markers. This step was performed using the Dynabeads Antibody Coupling Kit (Thermo Fisher Scientific). Magnetic beads (1.25 mg) containing epoxy groups were weighed and transferred into microcentrifuge tubes. The beads were suspended in 1 mL of 0.1 M sodium phosphate solution and vortexed. To remove any impurities from the beads, the tubes containing the suspended beads were placed on a magnet (DynaMag‐2 Magnet; Thermo Fisher Scientific) for 1 min, and the supernatant was removed. Next, the washed magnetic beads were coated with antibody (25 µL per antibody; 1 mg/mL). A 0.1 M sodium phosphate solution was added to adjust the final amount of the antibody‐added buffer to 250 µL. After adding Buffer C1 and vortexing, 250 µL of 3 M ammonium sulphate solution (Buffer C2) was added and gently vortexed. The mixture was then incubated overnight at 37°C with slow tilt rotation.

The following day, the mixture was washed by exploiting the magnetic properties of the beads. The tube was placed on a magnet, and the supernatant was removed. The beads were washed twice with 800 µL of PBS containing 0.1% bovine serum albumin (BSA). Next, the washing step using 800 µL of PBS with 1% BSA was performed twice. For the final wash, 800 µL of PBS containing 1% BSA was added and incubated for 15 min at room temperature. The immunomagnetic beads were resuspended in 500 µL PBS containing 1% BSA and stored at 4°C until use.

To isolate TB‐IEVs from the plasma using a bead‐based immunoprecipitation assay, 50 µL immunomagnetic beads was mixed with 50 µL plasma and 150 µL PBS containing 0.1% BSA. The mixture was then incubated for 1 h. After incubation, the beads were washed twice with 1 mL PBS containing 0.1% BSA and resuspended in 500 µL PBS containing 1% BSA. Bead‐captured EVs were stored at 4°C until downstream analysis.

### 3.7 NTA

EVs were characterized by NTA using a NanoSight LM10 (Malvern Instruments Ltd., Malvern, UK). Basic parameters, including size distribution and median values, were analysed using NTA software version 3.3 according to the manufacturer's instructions. The EV size and concentration were calculated based on the Stokes–Einstein equation by tracking the Brownian motion of particles captured using a laser‐illuminated camera.

### 3.8 TEM and SEM Analyses

EV size and morphology were confirmed using TEM. Samples were air‐dried on a copper grid with 200‐mesh carbon films (CF200‐cU; Electron Microscopy Science Inc., Hatfield, PA, USA). Grids were negatively stained with 2% (w/v) uranyl acetate for 5 min, dehydrated, and imaged using a transmission electron microscope (JEM‐2100 Plus; JEOL Ltd., Akishima, Tokyo, Japan).

To examine attachment of TB‐IEVs to antibody‐coated magnetic beads, samples were imaged using a scanning electron microscope (MERLIN; Carl Zeiss, Oberkochen, Germany). Beads with captured EVs were fixed with 2.5% glutaraldehyde in PBS for 30 min and washed twice with 100 µL PBS. The samples were then dehydrated in gradually increasing concentrations of ethanol (30%, 50%, 70%, 95% and 100%) for 10 min at each step. Samples were then coated with a thin gold film and imaged by SEM.

### 3.9 Fluorescence Analysis Using Confocal Microscopy

To visualize the EVs captured on TB‐IEV immunomagnetic beads, confocal microscopy was performed using fluorescence immunostaining. After TB‐IEV capture, bead‐bound EVs were incubated with PE‐Cy7–conjugated anti‐CD63 antibody and PerCP‐conjugated anti‐CD9 antibody (final volume: 200 µL in PBS with 0.1% BSA) for 30 min at room temperature in the dark. Bead–EV complexes were then washed three times with PBS containing 0.1% BSA to eliminate unbound antibodies and resuspended in 100 µL PBS.

For imaging analysis, 10 µL of the stained bead suspension was loaded onto a glass slide and sealed with a cover glass. Fluorescence images were acquired using a laser scanning confocal microscope (LSM 900; Carl Zeiss), with green and red signals representing CD63‐positive and CD9‐positive EVs, respectively. The number of fluorescent EV particles per field was quantified using ImageJ (National Institutes of Health), and the relative fluorescent particle counts of CD63 and CD9 were compared to determine the optimal TB‐IEV detection antibody.

### 3.10 Protein Isolation and Western Blotting Analysis

Proteins from EVs were lysed by adding 30 µL of 1 × RIPA lysis buffer (Thermo Fisher Scientific) and incubation for 15 min at 4°C.

Proteins (20 µg) were lysed with a non‐reducing lithium dodecyl sulphate sample buffer (Thermo Fisher Scientific) and boiled for 5 min at 100°C. The prepared protein samples were separated on 10% gels and transferred onto polyvinylidene difluoride membranes (Roche, Basel, Switzerland). Next, the membranes were submerged in 5% skim milk in PBST (PBS containing 0.5% Tween 20) for 90 min, followed by overnight incubation with primary antibodies (1:1000) at 4°C (Table S8). The immunoblotted membranes were washed with PBST and incubated with HRP‐conjugated secondary antibodies (1:3000) (Table S9). For visualization, the signals were enhanced using SuperSignal WestFemto (Thermo Fisher Scientific). Uncropped blots for western blot analysis are shown in Figures S7 and S8.

### 3.11 Flow Cytometry

Clinical evaluation of the isolated TB‐IEVs as potential diagnostic markers for TB was performed using flow cytometry. Immunobead‐bound TB‐IEVs (50 µL) were incubated with diluted anti‐CD63‐PE Cy7 (BD) for 30 min at 4°C in the dark. The captured TB‐IEVs were washed three times with 1 × PBS containing 0.1% BSA to remove nonspecific binding and then analysed using a FACS LSR II flow cytometer (BD Biosciences, Franklin Lakes, NJ, USA).

### 3.12 ELISA

Commercial ELISA kits were used to detect RAB8A, TYROBP, C3AR1, LILRA2 and LAM proteins (Table S10). As incubation times, washing steps and sample dilutions differed among kits, assays were performed according to the manufacturer's protocol. The absorbance was measured at 450 nm.

### 3.13 Numerical Simulation and Characterization

To evaluate the fluidic and magnetic performance of the 8‐channel microfluidic chip, numerical simulations were conducted using COMSOL Multiphysics (COMSOL Inc., USA). In CFD simulations, a constant volumetric flow rate of 1 mL/min was applied at the inlet to validate flow uniformity across the 8‐channel architecture. For the magnetic field analysis, a 3D model of the microwell array and external NdFeB magnets were constructed to determine the magnetic flux density ($\overset{⇀}{B}$). The magnetic flux density profile was specifically extracted along the a—a ′ section of the channel to assess the intensity distribution across the chambers. The magnetic force ($F_{m}$) acting on single microbead using Equation (1).

(1) $$F_{m}=\frac{1}{\mu_{0}}\Delta \chi \;V_{p}\overset{⇀}{B}\;\left(\nabla \cdot \overset{⇀}{B}\right)$$

where *μ_0_* is the permeability of free space, Δχ is the difference in magnetic susceptibility between the particle and the surrounding medium (dimensionless), *V_p_* is the volume of the magnetic microbeads and $\overset{⇀}{B}$ is the magnetic flux density.

Finally, the capture efficiency of the magnets was evaluated using magnetic beads at different concentrations (10^6^–10^9^ beads/mL). The magnetic beads were manually introduced through the inlet to be pre‐loaded into each chamber. The capture efficiency was quantified by controlling the placement time of the magnets and enumerating the number of beads before and after the capture process to determine the fraction of immobilized beads.

### 3.14 Fabrication of the Microfluidic Chip

The microfluidic chip was fabricated using conventional soft lithography. Briefly, a silicon wafer was spin‐coated with SU‐8 2100 photoresist (Kayaku Advanced Materials, USA) to produce a channel thickness of 500 µm and a chamber thickness of 1.5 mm. The wafer was patterned by UV exposure through a photomask, followed by development and post‐baking to obtain the master mold. PDMS (Sylgard 184, Dow Corning, USA) was prepared in a 10:1 (base:curing agent) ratio, poured onto the mold, degassed and cured at 95°C for 1 h. The cured PDMS replica was peeled off, and eight inlet/outlet ports were created using a 2 mm biopsy punch (Elveflow, France). Finally, the PDMS replica was irreversibly bonded to a glass substrate via oxygen plasma treatment (Femto Science, Korea). Upon completion of chip fabrication, a NdFeB magnet with dimensions identical to those of the chamber was placed beneath each chamber for magnetic bead capture.

### 3.15 Fluorescence Detection Using the Multichannel Chip

The multichannel chip was used to perform a fluorescence immunoassay and to evaluate its performance in clinical sample analysis. First, 2 µL of immunomagnetic beads were preloaded into each reaction chamber on the chip and immobilized at the chamber bottom using an external magnet underneath the chip. Clinical samples (10 µL) were mixed with 0.5 µL of CD63 fluorescent antibody and incubated at room temperature for 30 min in the dark. The sample–antibody mixture was then introduced through the chip inlet and evenly distributed across all eight chambers. Because the immunomagnetic beads were fixed using a magnet, they remained suspended within the reaction chamber, enabling sufficient binding interactions between the beads and target analytes in the sample. The reaction was conducted at room temperature for 30 min in the dark.

The chip was then placed on a magnet for 1 min to stabilize the magnetic beads. Washing buffer (1 × PBS) was injected through the inlet to rinse all eight chambers, and this step was repeated three times to remove unbound or nonspecific components. After washing, PBS was introduced again from the inlet to ensure that each chamber was filled with ∼5 µL of buffer. The chip was placed on an iBright Fluorescence Imaging System (Thermo Fisher Scientific) for fluorescence detection.

### 3.16 Statistical Analysis

Statistical analysis was performed using MeV 4.9 and GraphPad Prism software version 8.4.3 (GraphPad, La Jolla, CA, USA). Group comparisons were performed using the Mann–Whitney *U* test for two groups and the Kruskal–Wallis test with Dunn's post hoc test for three groups. Paired pre‐ and post‐treatment comparisons were performed using the Wilcoxon matched‐pairs signed‐rank test. For each participant, the combined TB‐IEV value was calculated as the arithmetic mean of the fluorescence intensities measured for RAB8A, TYROBP, C3AR1 and LILRA2; this composite was prespecified and equally weighted, without statistical optimization of marker weighting or selection. Receiver operating characteristic (ROC) curves were generated for each individual marker and for the combined TB‐IEV value, and optimal cutoffs were determined using the Youden index. Areas under the curve (AUCs) are reported with 95% confidence intervals, together with the corresponding sensitivity and specificity. TB‐IEV positivity was defined as a combined TB‐IEV value exceeding the ROC‐derived cutoff obtained from the ATB‐versus‐HC comparison (Table 1). *p* values were not adjusted for multiple comparisons and are therefore considered descriptive. Statistical significance was set at *p* values < 0.05.

## 4 Discussion

In the landscape of TB diagnostics, this study introduced a targeted EV‐based strategy that leverages immune cell‐associated EV surface markers and a rapid multichannel microfluidic platform, providing a biologically informed approach with translational potential for TB detection. Although traditional diagnostic methods for ATB rely on lower respiratory tract samples and have limitations, including prolonged processing times and susceptibility to false results (Steingart et al. 2006; Mukherjee et al. 2023), the emergence of EVs as diagnostic candidates offers a promising alternative (Mehaffy et al. 2022).

EVs, ranging from 50 to 200 nm in size, are secreted by various cell types and ubiquitously found in body fluids, including blood (Jayaseelan 2020). Their cargo, comprising proteins, lipids and nucleic acids, reflects the physiological state of parent cells, making them attractive diagnostic candidates (Han et al. 2022; Kalluri and LeBleu 2020). Notably, encapsulation within a phospholipid membrane protects these biomarkers from degradation by proteinases and nucleases, ensuring high stability in circulation (Keller et al. 2011; Tkach and Théry 2016). A key distinction of our study is shifting the diagnostic focus from the pathogen to the host immune response. Unlike previous studies that focused on intravesicular cargo or general EV membrane proteins (Kim et al. 2021; Park et al. 2021; He et al. 2023), our study specifically targeted TB‐IEVs, which are EVs carrying surface proteins associated with innate immune cell responses to MTB infection. Focusing on surface‐exposed markers enables direct, lysis‐free capture, preserves the integrity of vesicles and simplifies the detection workflow. More importantly, by targeting the systemic host response to infection rather than the pathogen itself, our approach may reduce dependence on direct pathogen detection in respiratory specimens, which can yield negative results when bacillary burden is low or unevenly distributed.

By integrating the principles of host‐pathogen interactions and membrane biogenesis, we identified four surface‐enriched TB‐IEV markers: RAB8A, TYROBP, C3AR1 and LILRA2. Importantly, these are not canonical markers for adaptive immune cells such as T cells, but rather proteins predominantly expressed on myeloid cells actively involved in the innate immune response. The biological functions of these markers provide relevant mechanistic context for their association with host immune responses during MTB infection. To clarify the biological basis of the selected panel, the established functions of TB‐IEV markers and the evidence linking them to TB or mycobacterial infection are summarized in Table 2. The TB‐IEV markers represent distinct but complementary components of the innate immune response to mycobacterial infection. RAB8A, a substrate for LRRK2, contributes to cell morphogenesis (Peränen 2011), receptor recycling and intracellular transport (Kobayashi et al. 2014; Wu et al. 2023). In macrophages, pathogen‐ or sterile‐induced endomembrane damage activates LRRK2, which recruits RAB8A and ESCRT‐III machinery to damaged endolysosomes and favours membrane repair over lysophagy (Herbst et al. 2020). LRRK2 also recruits RAB8A to maturing phagosomes in human macrophages (Lee et al. 2020). This membrane‐repair function is particularly relevant to mycobacterial infection because *M. tuberculosis* can induce ESX‐1‐dependent phagosomal membrane damage and, under certain conditions, gain access to the host‐cell cytosol (van der Wel et al. 2007; Simeone et al. 2012). Although a direct role for RAB8A in *M. tuberculosis*‐containing EV formation has not been established, mycobacterial phagosomes show selective remodelling of Rab GTPase recruitment (Seto et al. 2011; Bussi and Gutierrez 2019), and RAB8A regulates macrophage Toll‐like‐receptor signalling, PI3K‐gamma‐AKT‐mTOR activation and the balance between proinflammatory cytokines and IL‐10 (Wall et al. 2019, 2017). RAB8A‐dependent unconventional and vesicular secretion has also been demonstrated in inflammatory cell models (Chen et al. 2017). Collectively, these observations connect RAB8A to the phagosomal membrane remodelling, innate signalling and secretory pathways that are perturbed during mycobacterial infection.

**TABLE 2:** Biological rationale for the selected TB‐IEV markers in the context of host immune responses to tuberculosis.

Marker	Cellular context	Biological functions	Evidence related to TB or mycobacterial infection	References
RAB8A	Macrophages; endolysosomal and phagosomal compartments	Cell morphogenesis; receptor recycling; intracellular transport; endomembrane repair; TLR signalling; cytokine regulation; vesicular secretion	LRRK2‐dependent recruitment of RAB8A and ESCRT‐III to damaged endolysosomes; RAB8A recruitment to maturing phagosomes; selective remodelling of Rab GTPase recruitment in mycobacterial phagosomes; PI3Kγ–AKT–mTOR‐dependent signalling and regulation of the proinflammatory cytokine–IL‐10 balance	(Peränen 2011; Kobayashi et al. 2014; Wu et al. 2023: Herbst et al. 2020; Lee et al. 2020; van der Wel et al. 2007; Simeone et al. 2012; Bussi and Gutierrez 2019; Wall et al. 2019; Wall et al. 2017; Chen et al. 2017)
TYROBP	Lung macrophages; antigen‐presenting cells	Inflammatory modulation; DAP12‐associated myeloid signalling; immunoregulatory responses	Regulation of DAP12‐associated receptors during pulmonary mycobacterial infection; IRAK‐M‐ and IL‐10‐associated immunoregulatory phenotype; delayed Th1 activation; TREM2 recognition of mycolic‐acid‐containing lipids; enhanced macrophage activation and mycobacterial elimination following disruption of TREM2–DAP12 signalling	(Bouchon et al. 2001; Hiremath et al. 2020; Aoki et al. 2004; Jeyanathan et al. 2014; Iizasa et al. 2021; Divangahi et al. 2007)
C3AR1	Macrophages/monocytes; neutrophils; pleural mesothelial cells; CD14^+^CD16^+^ nonclassical monocytes	Cellular metabolism; innate immune responses; immunomodulation; regulation of neutrophil function	Complement C3 binding to M. tuberculosis in the pulmonary environment; C3a–C3AR1‐dependent CCL2, CCL7, and CX3CL1 production; recruitment of CD14^+^CD16^+^ nonclassical monocytes; inflammatory cytokine production and Th17 expansion	(Wagner and Frank 2010; Gedam and Zheng 2024; Corcoran and Napier 2022; Brennan et al. 2019; Ferguson et al. 2004; Luo et al. 2019; Deng et al. 2021)
LILRA2	Monocytes; macrophages; neutrophils; other innate immune cells	Microbial danger sensing; inflammatory cytokine regulation; TLR4‐response modulation; inhibition of Fc‐receptor‐dependent phagocytosis; regulation of antigen‐presenting‐cell differentiation	Recognition of immunoglobulins cleaved by microbial proteases; impaired monocyte‐to‐dendritic‐cell differentiation; reduced presentation of mycobacterial antigens; enrichment in smear‐positive TB associated with higher bacterial burden and enhanced innate immune activation	(Hirayasu et al. 2016; Yamazaki et al. 2020; Lu et al. 2012; Lee et al. 2007; Ju et al. 2023)

Abbreviations: C3AR1, complement C3a receptor 1; DAP12, DNAX‐activating protein 12; ESCRT, endosomal sorting complexes required for transport; LILRA2, leukocyte immunoglobulin‐like receptor A2; LRRK2, leucine‐rich repeat kinase 2; RAB8A, Ras‐related protein Rab‐8A; TLR, toll‐like receptor; TYROBP, TYRO protein tyrosine kinase‐binding protein.

TYROBP (DAP12) functions as a key inflammatory modulator (Bouchon et al. 2001; Hiremath et al. 2020; Aoki et al. 2004). Pulmonary mycobacterial infection differentially regulates DAP12‐associated receptors in lung macrophages, placing this signalling module within the host antimicrobial response (Jeyanathan et al. 2014). Following MTB infection, DAP12 interacts with signalling kinases to induce an immunosuppressive phenotype in antigen‐presenting cells, activating TH1 cells via IL‐10 (Iizasa et al. 2021). Furthermore, the DAP12‐associated receptor TREM2 directly recognizes nonglycosylated mycobacterial mycolic‐acid‐containing lipids and recruits mycobacterium‐permissive macrophages; disruption of TREM2‐DAP12 signalling enhances macrophage activation and accelerates mycobacterial elimination. DAP12 can also restrain excessive type 1 immunity and tissue immunopathology during intracellular infection (Divangahi et al. 2007). Thus, increased EV‐associated TYROBP may reflect an active myeloid immunoregulatory program rather than simply increased abundance of a generic leukocyte protein.

C3AR1, a receptor for anaphylatoxin C3a, is a key proinflammatory reactant involved in cellular metabolism and innate immune responses (Wagner and Frank 2010; Gedam and Zheng 2024; Corcoran and Napier 2022). The C3a/C3AR1 axis exerts immunomodulatory effects and may attenuate neutrophil function (Brennan et al. 2019). Complement C3 can bind M. tuberculosis in the human pulmonary environment, providing a direct interface between the bacillus and complement pathways (Ferguson et al. 2004). In patients with tuberculous pleural effusion, the complement system is strongly activated, and C3a‐C3AR1 signalling stimulates pleural mesothelial cells to produce CCL2, CCL7 and CX3CL1, thereby recruiting CD14+CD16+ nonclassical monocytes into the pleural space (Luo et al. 2019). Anaphylatoxin‐conditioned monocytes also produce inflammatory cytokines and acquire costimulatory phenotypes that promote Th17 expansion (Deng et al. 2021). Collectively, these findings suggest that increased EV‐associated C3AR1 may reflect complement‐driven recruitment and activation of myeloid cells during pulmonary tuberculosis.

LILRA2 extends the panel to microbial danger sensing and regulation of antigen‐presenting‐cell function. LILRA2 is an activating receptor expressed on monocytes, macrophages, neutrophils and other innate immune cells. It detects immunoglobulins whose N termini have been cleaved by microbial proteases, allowing myeloid cells to sense host proteins modified during bacterial or fungal infection (Hirayasu et al. 2016; Yamazaki et al. 2020). LILRA2 stimulation induces a distinct inflammatory cytokine program, modulates TLR4 responses and inhibits Fc‐receptor‐dependent phagocytosis in human monocytes (Lu et al. 2012). It also impairs monocyte‐to‐dendritic‐cell differentiation and reduces presentation of mycobacterial antigens to T cells while maintaining inflammatory cytokine production (Lee et al. 2007). Supporting its relevance to pulmonary TB, quantitative plasma proteomics identified LILRA2 among proteins enriched in smear‐positive compared with smear‐negative TB and healthy controls; this protein pattern was associated with higher bacterial burden, altered antigen‐presenting‐cell activity and stronger innate immune activation (Ju et al. 2023). The enrichment of these proteins on circulating EVs suggests that TB‐IEVs serve as minimally invasive, blood‐based indicators of the complex immune dynamics occurring in the lung, offering a real‐time snapshot that is otherwise inaccessible without invasive procedures.

To translate these biological insights into clinical practice, we developed a customized multichannel microfluidic chip. CD9, CD63 and CD81 were evaluated as canonical tetraspanin markers of EV‐enriched material (Hallal et al. 2022; Pegtel and Gould 2019). Among the tested fluorescent detection antibodies, CD63 produced stronger labelling than CD9 in the marker‐captured EV preparations and was therefore selected for EV detection. This platform enables simultaneous sample processing and rapid EV capture, markedly reducing assay time and improving reproducibility relative to conventional single‐channel formats. Flow cytometric validation and chip‐based assays demonstrated that TB‐IEVs provided superior diagnostic accuracy compared with total EVs captured using canonical markers (CD9, CD63 and CD81), achieving high sensitivity and specificity. Importantly, the discriminatory performance of the TB‐IEV signature was further evaluated in 40 patients with other respiratory diseases (ORDs), most commonly pneumonia, bronchitis and chronic obstructive pulmonary disease, among other diverse conditions. The combined signature distinguished ATB from this heterogeneous ORD cohort with an AUC of 0.95, indicating that its discriminatory performance extended beyond the comparison between ATB and healthy individuals. The multichannel chip system highlights the translational potential of this method by enabling rapid, efficient diagnosis that is readily adaptable to clinical workflows (Lehnert and Gijs 2024).

The most notable clinical impact of this study was the ability of the TB‐IEV signature to provide complementary diagnostic information in patients with established ATB who had negative results on individual conventional diagnostic tests. The multichannel chip showed a high positivity rate in this ATB cohort, including among subgroups with negative results on individual conventional diagnostic tests. Importantly, the patients included in these subgroup analyses had an ATB diagnosis established independently of the TB‐IEV assay based on microbiological, pathological and/or clinical‐radiological criteria; therefore, TB‐IEV positivity itself was not used to establish the diagnosis. Moreover, patients classified as PCR‐negative, chest X‐ray‐negative, or culture‐negative were not necessarily negative across all conventional diagnostic modalities simultaneously. Rather, these subgroup analyses demonstrate that the TB‐IEV signature retained a high detection rate when an individual conventional test yielded a negative result. Patient‐level conventional diagnostic findings for these cases are provided in Table S6. Accordingly, the chip‐based TB‐IEV assay may complement existing diagnostic approaches, particularly when individual pathogen‐based tests yield negative results. Although some patients with limited microbiological evidence could potentially represent earlier or subclinical disease states, the present study was not designed to distinguish subclinical TB from clinically manifest ATB. Further prospective studies incorporating longitudinal microbiological, radiological and clinical assessments will be required to determine whether the TB‐IEV signature can identify such disease states. Furthermore, the dynamic reduction in TB‐IEV levels following anti‐TB treatment supports the potential utility of this signature for treatment‐response monitoring (Samanta et al. 2018; Kumar et al. 2024).

Despite these promising findings, our study has several limitations. First, although the study employed a sequential design encompassing transcriptomic discovery, biomarker validation and microfluidic device evaluation, the sample size within each clinical stage remained relatively limited. The additional evaluation in 40 patients with heterogeneous ORDs broadened the assessment beyond healthy controls and provided evidence of discrimination from clinically relevant non‐TB respiratory diseases. Nevertheless, these findings require confirmation in larger, prospectively enrolled, independent multicenter cohorts. Such studies should include important TB mimics not represented in the present cohort, particularly NTM pulmonary disease, fungal pulmonary infections and sarcoidosis, and should establish robust diagnostic thresholds and generalizability across diverse populations. Second, the cellular source and packaging mechanisms of the four proteins remain unresolved. Only a small fraction of circulating monocytes or neutrophils would be expected to be directly infected with MTB; therefore, the circulating EV signals detected in this study are unlikely to originate exclusively from directly infected blood cells. These EVs may also be released from activated circulating myeloid cells or tissue‐resident immune cells responding to MTB infection, while systemic activation, altered haematopoiesis, or secondary inflammatory signalling may further contribute to the observed signature. Future cell‐type‐specific EV tracing and paired cellular/EV profiling will be required to define the cellular origins of these signals. Third, although all eight longitudinal participants showed negative microbiological and chest X‐ray evaluations at T6, the treatment‐associated EV‐marker decreases require confirmation in larger cohorts with serial sampling, durable clinical outcomes and relapse surveillance. Fourth, because the current assay requires only 10 µL of plasma, finger‐prick blood sampling represents a promising option for future POC application and could reduce dependence on venipuncture. However, the present study used venous plasma, and differences in capillary‐plasma preparation, sample composition, matrix effects, EV recovery and equivalence to venous plasma require dedicated validation. Finally, although the current chip is a prototype, future efforts should focus on mass production and automation to facilitate its deployment in resource‐limited settings. Beyond TB, this “immune‐EV profiling” concept offers a versatile framework applicable to other infectious diseases where pathogen isolation is difficult.

In conclusion, this study demonstrates the potential of an immune cell‐associated EV signature integrated with multichannel microfluidics for rapid, low‐volume plasma‐based ATB detection. The TB‐IEV signature discriminated ATB from both HCs and a heterogeneous cohort of ORDs and provided complementary diagnostic information in patients with established ATB who had negative results on individual conventional tests. Treatment‐associated changes in TB‐IEV signals further support its potential application for treatment‐response monitoring. Although larger prospective multicentre validation and further investigation of the biological origins and mechanisms of these EV‐associated markers are required, this study provides a translational framework for the development of host‐response‐based, non‐sputum diagnostic approaches for TB.

## Author Contributions

**Eun Ju Lee**: methodology, investigation, formal analysis, writing – original draft, writing – review and editing, visualization. **Cheng Nie**: methodology, investigation, visualization, writing – original draft, writing – review and editing, formal analysis. **Sunyoung Park**: conceptualization, methodology, investigation, visualization, writing – original draft, writing – review and editing, formal analysis. **Yun‐Jeong Kang**: methodology, investigation, writing – review and editing. **Minji Kang**: investigation, methodology, writing – review and editing. **Jake Whang**: investigation, methodology, writing – review and editing. **Go‐Eun Choi**: investigation, methodology, writing – review and editing. **Hyejon Lee**: investigation, methodology, writing – review and editing. **San‐Nae Cho**: investigation, methodology, writing – review and editing. **Kyung‐A. Hyun**: conceptualization, methodology, writing – original draft, writing – review and editing, supervision. **Sunghyun Kim**: conceptualization, methodology, writing – original draft, writing – review and editing, supervision, funding acquisition. **Hyo‐Il Jung**: conceptualization, methodology, writing – original draft, writing – review and editing, supervision. **Jungho Kim**: conceptualization, methodology, writing – original draft, writing – review and editing, funding acquisition, supervision.

## Funding

This study was supported by the Basic Science Research Program through the National Research Foundation of Korea (NRF), funded by the Ministry of Science, ICT and Future Planning (RS‐2023‐00211685, RS‐2024‐00463065, RS‐2026‐25493296), BB21 plus funded by Busan Metropolitan City and Busan Techno Park.

## Conflicts of Interest

H.‐I. Jung is affiliated with The DABOM Inc. The company had no role in study design, data collection, analysis, interpretation, or the decision to publish. The other authors declare no competing interests.

## Supporting information




**Supporting information**: jev270395‐sup‐0001‐SuppMat.docx

## Data Availability

The sequencing datasets generated for this study are available from the Gene Expression Omnibus (GEO) at https://www.ncbi.nlm.nih.gov/geo/ (accession number: GSE279063). All the datasets generated in this study are included in the article/supplementary material section.
