# Supplementary material for: Microfluidic Profiling of an Immune Cell‐Associated Extracellular Vesicle Signature for Active Tuberculosis Diagnosis and Treatment Monitoring
Source: J Extracell Vesicles. 2026 Nov 3;15(11):e70395. doi: 10.1002/jev2.70395 (PMC13730261; doi:10.1002/jev2.70395)
Supplement: Supplementary file 1 [file JEV2-15-e70395-s001.docx]

**Supplemental Data**

**Microfluidic Profiling of an Immune Cell-Associated Extracellular Vesicle Signature for Active Tuberculosis Diagnosis and Treatment Monitoring**

Eun Ju Lee^1,2,†^, Cheng Nie^3,4†^, Sunyoung Park^5,†^, Yun-Jeong Kang^6^, Minji Kang^7^, Jake Whang^7^, Go-Eun Choi^1,2^, Hyejon Lee^8^, San-Nae Cho^8,9^, Kyung-A Hyun^10,11*^, Sunghyun Kim^1,2,*^, Hyo-Il Jung^3,12,13,*^, Jungho Kim^1,2,*^

^1^Department of Clinical Laboratory Science, College of Health Sciences, Catholic University of Pusan, Busan 46252, Republic of Korea

^2^Next-Generation Industrial Field-Based Specialist Program for Molecular Diagnostics, Brain Busan 21 Plus Project, Graduate School, Catholic University of Pusan, Busan 46252, Republic of Korea

^3^School of Mechanical Engineering, Yonsei University, Seoul 03772, Republic of Korea

^4^School of Mechanical and Electrical Engineering, Zaozhuang University, 1 Bei’an Road, Zaozhuang, Shandong 277160, China

^5^Department of Biomedical Technology, Kangwon National University, Chuncheon 24341, Republic of Korea

^6^Department of Biomedical Laboratory Science, Dong-Eui Institute of Technology, Busan 47230, Republic of Korea

^7^Korea Mycobacterium Resource Center (KMRC), Department of Research and Development, The Korean Institute of Tuberculosis, Osong 28158, Republic of Korea

^8^Clinical Vaccine Research Section, International Tuberculosis Research Center, Changwon 51755, Republic of Korea

^9^Institute of Immunology and Immunological Disease, Yonsei University College of Medicine, Seoul 03772, Republic of Korea

^10^School of Biopharmaceutical and Medical Sciences, Sungshin Women’s University, Seoul 01133, Republic of Korea

^11^Basic Science Institute, Sungshin Women’s University, Seoul 01133, Republic of Korea

^12^The DABOM Inc., 50 Yonsei-ro, Seodaemun-gu, Seoul 03722, Republic of Korea.

^13^The Institute of Biotechnology and Genetic Engineering, Chulalongkorn University, Bangkok 10330, Thailand

**Table S1. List of 297 differentially expressed genes identified by comparison between the ATB and HC groups**

| **No.** | **Gene symbol** | **HC mean** | **HC**  **SD** | **ATB mean** | **ATB**  **SD** | **Log_2_**  **(Fold change)** | **Adjusted**  ***p*-value** |
| --- | --- | --- | --- | --- | --- | --- | --- |
| 1 | PDCD4-AS1 | -0.7142172 | 0.6458549 | 0.8332533 | 0.61081976 | 1.5474705 | 2.13E-06 |
| 2 | ARPC1A | -0.71158314 | 0.56787574 | 0.83018035 | 0.70378345 | 1.54176349 | 4.94E-06 |
| 3 | PPID | -0.6941939 | 0.44888243 | 0.80989295 | 0.84006315 | 1.50408685 | 4.32E-05 |
| 4 | RAB8A | -0.64980453 | 0.40491 | 0.75810534 | 0.9563423 | 1.40790987 | 3.12E-04 |
| 5 | DAZAP2 | -0.6467053 | 0.7324873 | 0.7544895 | 0.6966652 | 1.4011948 | 4.75E-05 |
| 6 | LILRA6 | -0.6458145 | 0.46430737 | 0.75345016 | 0.93157387 | 1.39926466 | 2.71E-04 |
| 7 | DYNLRB1 | -0.6413088 | 0.6653404 | 0.7481937 | 0.7844966 | 1.3895025 | 9.05E-05 |
| 8 | LSM10 | -0.6386215 | 0.5178523 | 0.7450585 | 0.9116772 | 1.38368 | 2.65E-04 |
| 9 | MAX | -0.6337676 | 0.41686934 | 0.7393956 | 0.97966266 | 1.3731632 | 4.83E-04 |
| 10 | HNRNPH3 | -0.63218516 | 0.44396433 | 0.7375495 | 0.96834815 | 1.36973466 | 4.89E-04 |
| 11 | CTSA | -0.6266614 | 0.2577777 | 0.7311049 | 1.0541744 | 1.3577663 | 9.42E-04 |
| 12 | MAGOH | -0.6248724 | 0.5832973 | 0.7290178 | 0.89100635 | 1.3538902 | 2.76E-04 |
| 13 | ABCA1 | -0.619769 | 0.46837175 | 0.72306377 | 0.97685575 | 1.34283277 | 5.69E-04 |
| 14 | LOC728084 | -0.619654 | 0.7530494 | 0.72292966 | 0.7373661 | 1.34258366 | 1.32E-04 |
| 15 | FRAT2 | -0.61396295 | 0.73826444 | 0.7162902 | 0.76754546 | 1.33025315 | 1.69E-04 |
| 16 | TYROBP | -0.61393803 | 0.6173585 | 0.7162611 | 0.88482404 | 1.33019913 | 3.26E-04 |
| 17 | RNASEK | -0.6123777 | 0.57251275 | 0.71444076 | 0.92263365 | 1.32681846 | 4.65E-04 |
| 18 | S100A11 | -0.61202425 | 0.4479971 | 0.71402836 | 1.0013088 | 1.32605261 | 8.27E-04 |
| 19 | ACTR2 | -0.6113992 | 0.66351324 | 0.71329904 | 0.84948546 | 1.32469824 | 2.91E-04 |
| 20 | RHBDD2 | -0.6113567 | 0.6856681 | 0.71324944 | 0.82851875 | 1.32460614 | 2.52E-04 |
| 21 | PKMYT1 | -0.6106597 | 0.5524433 | 0.7124364 | 0.94006884 | 1.3230961 | 5.03E-04 |
| 22 | IFITM2 | -0.6103154 | 0.44008473 | 0.71203464 | 1.0083076 | 1.32235004 | 8.70E-04 |
| 23 | ZFAND3 | -0.60691446 | 0.6235199 | 0.70806694 | 0.89304197 | 1.3149814 | 4.01E-04 |
| 24 | C3AR1 | -0.60599995 | 0.719696 | 0.70699996 | 0.8049279 | 1.31299991 | 2.55E-04 |
| 25 | PTGER3 | -0.6042642 | 0.46302834 | 0.70497483 | 1.0062124 | 1.30923903 | 9.88E-04 |

(Table S1 continues on the following page).

**Table S1.** (continued)

| **No.** | **Gene symbol** | **HC mean** | **HC**  **SD** | **ATB mean** | **ATB**  **SD** | **Log_2_**  **(Fold change)** | **Adjusted**  ***p*-value** |
| --- | --- | --- | --- | --- | --- | --- | --- |
| 26 | GPR27 | -0.60399806 | 0.5290358 | 0.70466447 | 0.96745026 | 1.30866253 | 7.07E-04 |
| 27 | DEDD2 | -0.60361594 | 0.80329263 | 0.7042186 | 0.710912 | 1.30783454 | 2.06E-04 |
| 28 | PBX2 | -0.5974621 | 0.7548073 | 0.6970392 | 0.7842565 | 1.2945013 | 2.88E-04 |
| 29 | ST6GALNAC2 | -0.5959762 | 0.79516333 | 0.6953056 | 0.738935 | 1.2912818 | 2.74E-04 |
| 30 | GABARAPL1 | -0.5903605 | 0.88808054 | 0.6887539 | 0.6160819 | 1.2791144 | 2.58E-04 |
| 31 | CEP63 | -0.5884525 | 0.6325299 | 0.6865279 | 0.9192419 | 1.2749804 | 6.80E-04 |
| 32 | MYLIP | -0.58431137 | 0.45212135 | 0.6816965 | 1.0438663 | 1.26600787 | 0.001603461 |
| 33 | ANP32E | -0.5835697 | 0.5362231 | 0.6808314 | 0.9969003 | 1.2644011 | 0.001188399 |
| 34 | LILRA2 | -0.5834126 | 0.84828794 | 0.68064815 | 0.69548744 | 1.26406075 | 3.65E-04 |
| 35 | CEBPD | -0.583011 | 0.49199277 | 0.6801794 | 1.0243804 | 1.2631904 | 0.001411985 |
| 36 | RAB7A | -0.5808429 | 0.7618294 | 0.67765015 | 0.81022966 | 1.25849305 | 5.23E-04 |
| 37 | FLI1 | -0.5791808 | 0.665541 | 0.6757109 | 0.9078641 | 1.2548917 | 8.36E-04 |
| 38 | C19orf43 | -0.5770305 | 0.4354205 | 0.6732022 | 1.0632457 | 1.2502327 | 0.001917395 |
| 39 | MYD88 | -0.5739787 | 0.4663473 | 0.6696418 | 1.0522444 | 1.2436205 | 0.001998847 |
| 40 | LOC441155 | -0.5731656 | 0.61068094 | 0.6686931 | 0.96232784 | 1.2418587 | 0.001161803 |
| 41 | CXCR2P1 | -0.5711757 | 0.34578565 | 0.66637164 | 1.1098576 | 1.23754734 | 0.002973532 |
| 42 | NRM | -0.56924504 | 0.82485914 | 0.6641192 | 0.7583288 | 1.23336424 | 6.06E-04 |
| 43 | RAC1 | -0.56922495 | 0.5534429 | 0.6640958 | 1.0085812 | 1.23332075 | 0.001652388 |
| 44 | AKIRIN1 | -0.5663167 | 0.5807832 | 0.6607028 | 0.9948337 | 1.2270195 | 0.001565067 |
| 45 | LAPTM5 | -0.5662931 | 0.75561196 | 0.6606754 | 0.8447767 | 1.2269685 | 8.17E-04 |
| 46 | CITED2 | -0.56604725 | 0.59569174 | 0.66038847 | 0.9847878 | 1.22643572 | 0.001546956 |
| 47 | MS4A2 | -0.56586367 | 0.7076273 | 0.6601743 | 0.8932903 | 1.22603797 | 0.001037104 |
| 48 | ERGIC1 | -0.5653726 | 0.84559745 | 0.6596013 | 0.7390788 | 1.2249739 | 6.51E-04 |
| 49 | ADAM9 | -0.564175 | 0.39826137 | 0.6582042 | 1.0988897 | 1.2223792 | 0.002919324 |
| 50 | PPIF | -0.5641591 | 0.7559277 | 0.6581856 | 0.84837264 | 1.2223447 | 8.69E-04 |
| 51 | COTL1 | -0.5628532 | 0.804199 | 0.65666205 | 0.7967389 | 1.21951525 | 7.69E-04 |

(Table S1 continues on the following page).

**Table S1.** (continued)

| **No.** | **Gene symbol** | **HC mean** | **HC**  **SD** | **ATB mean** | **ATB**  **SD** | **Log_2_**  **(Fold change)** | **Adjusted**  ***p*-value** |
| --- | --- | --- | --- | --- | --- | --- | --- |
| 52 | DLD | -0.56229126 | 0.67935663 | 0.65600646 | 0.92489 | 1.21829772 | 0.001285996 |
| 53 | TLE4 | -0.5598101 | 0.6469967 | 0.65311176 | 0.95594114 | 1.21292186 | 0.00155149 |
| 54 | LOC100128233 | -0.559651 | 1.0919046 | 0.652926 | 0 | 1.212577 | 0.001130256 |
| 55 | CALM3 | -0.5594949 | 0.483971 | 0.6527441 | 1.0642818 | 1.212239 | 0.002695838 |
| 56 | SERTAD3 | -0.5585764 | 0.49693787 | 0.65167254 | 1.0585347 | 1.21024894 | 0.002457209 |
| 57 | SHKBP1 | -0.55819017 | 0.6690432 | 0.6512218 | 0.9404936 | 1.20941197 | 0.001451214 |
| 58 | TOP2A | -0.55810016 | 0.7669038 | 0.65111685 | 0.8477812 | 1.20921701 | 0.001010122 |
| 59 | TMEM11 | -0.55761045 | 0.8423418 | 0.6505456 | 0.7594529 | 1.20815605 | 8.27E-04 |
| 60 | KCTD2 | -0.5575987 | 0.7402676 | 0.65053177 | 0.87619656 | 1.20813047 | 0.001145506 |
| 61 | JUNB | -0.5563764 | 0.61799645 | 0.64910585 | 0.9837414 | 1.20548225 | 0.001899843 |
| 62 | PIAS1 | -0.55602884 | 0.62100124 | 0.6487003 | 0.98204553 | 1.20472914 | 0.001762277 |
| 63 | OAZ2 | -0.5556219 | 0.8127015 | 0.6482255 | 0.8005302 | 1.2038474 | 9.33E-04 |
| 64 | RAD51 | -0.55495006 | 0.67174566 | 0.6474417 | 0.9434997 | 1.20239176 | 0.0015693 |
| 65 | TUBA1C | -0.55388486 | 0.40584204 | 0.6461989 | 1.1099912 | 1.20008376 | 0.00357328 |
| 66 | DUSP10 | -0.55380327 | 0.6336318 | 0.6461038 | 0.9759594 | 1.19990707 | 0.001830358 |
| 67 | SEMA3C | -0.5527245 | 0.8476489 | 0.6448453 | 0.76231825 | 1.1975698 | 9.42E-04 |
| 68 | PRR11 | -0.55270493 | 0.38487867 | 0.6448224 | 1.1203887 | 1.19752733 | 0.003706292 |
| 69 | RAB1B | -0.5525094 | 0.7811323 | 0.6445945 | 0.84251046 | 1.1971039 | 0.001148984 |
| 70 | REEP4 | -0.5523682 | 0.6405053 | 0.64442956 | 0.97289467 | 1.19679776 | 0.001878276 |
| 71 | NUMB | -0.55209005 | 0.7734648 | 0.644105 | 0.8515801 | 1.19619505 | 0.001178866 |
| 72 | CXCR2 | -0.55150014 | 0.62899625 | 0.64341676 | 0.9830737 | 1.1949169 | 0.001946406 |
| 73 | GCSAML | -0.54951113 | 0.5495737 | 0.6410964 | 1.0407194 | 1.19060753 | 0.002609138 |
| 74 | ASGR2 | -0.54891616 | 0.58826315 | 0.64040214 | 1.0163034 | 1.1893183 | 0.002341651 |
| 75 | CKAP2L | -0.54804784 | 0.75378275 | 0.63938916 | 0.8791907 | 1.187437 | 0.001444823 |
| 76 | RAB27A | -0.54770595 | 0.58972436 | 0.6389903 | 1.0171028 | 1.18669625 | 0.002403568 |
| 77 | RILPL2 | -0.54746336 | 0.71040213 | 0.6387073 | 0.9218501 | 1.18617066 | 0.001673684 |

(Table S1 continues on the following page).

**Table S1.** (continued)

| **No.** | **Gene symbol** | **HC mean** | **HC**  **SD** | **ATB mean** | **ATB**  **SD** | **Log_2_**  **(Fold change)** | **Adjusted**  ***p*-value** |
| --- | --- | --- | --- | --- | --- | --- | --- |
| 78 | ARHGDIB | -0.5472454 | 0.7981981 | 0.63845295 | 0.83302766 | 1.18569835 | 0.001215257 |
| 79 | LINC00528 | -0.54705614 | 0.45271343 | 0.63823205 | 1.0978404 | 1.18528819 | 0.003577231 |
| 80 | CD68 | -0.5460741 | 0.5028595 | 0.63708645 | 1.0731183 | 1.18316055 | 0.003198488 |
| 81 | MAB21L3 | -0.54569495 | 0.4861837 | 0.6366441 | 1.0826893 | 1.18233905 | 0.00358412 |
| 82 | MYBPC3 | -0.545607 | 0.71606827 | 0.6365414 | 0.91970176 | 1.1821484 | 0.001739059 |
| 83 | CSNK1G2 | -0.54503316 | 0.59542656 | 0.63587195 | 1.0171359 | 1.18090511 | 0.002538319 |
| 84 | SMC4 | -0.5426699 | 0.57919323 | 0.6331148 | 1.0315952 | 1.1757847 | 0.002943157 |
| 85 | DNTTIP1 | -0.54176104 | 0.6816074 | 0.63205457 | 0.9561932 | 1.17381561 | 0.002141877 |
| 86 | LILRB3 | -0.5396021 | 0.8287616 | 0.62953573 | 0.8112189 | 1.16913783 | 0.001413045 |
| 87 | CTRL | -0.5393795 | 0.47676343 | 0.62927604 | 1.0962819 | 1.16865554 | 0.004099146 |
| 88 | IMPA2 | -0.5380441 | 0.7462165 | 0.62771803 | 0.903521 | 1.16576213 | 0.001892536 |
| 89 | H3F3AP4 | -0.537947 | 0.36862388 | 0.6276048 | 1.1463556 | 1.1655518 | 0.0055119 |
| 90 | APP | -0.5378757 | 0.6384022 | 0.6275217 | 0.9966311 | 1.1653974 | 0.002648614 |
| 91 | PITPNA | -0.5370254 | 0.6056197 | 0.62652963 | 1.0217588 | 1.16355503 | 0.0030033 |
| 92 | ARRB2 | -0.5368809 | 0.62315315 | 0.6263611 | 1.009434 | 1.163242 | 0.00295516 |
| 93 | PANX2 | -0.5357667 | 0.8403338 | 0.62506115 | 0.8041284 | 1.16082785 | 0.00153256 |
| 94 | ALDH4A1 | -0.5346121 | 0.7110332 | 0.6237141 | 0.941857 | 1.1583262 | 0.002297783 |
| 95 | PRKAG2 | -0.53459615 | 0.35124078 | 0.62369555 | 1.1570783 | 1.1582917 | 0.00590556 |
| 96 | SPHK1 | -0.5340006 | 0.46508127 | 0.6230008 | 1.1093953 | 1.1570014 | 0.004596626 |
| 97 | PANX1 | -0.5328507 | 0.79595214 | 0.6216591 | 0.8608369 | 1.1545098 | 0.001886445 |
| 98 | HIF1A-AS2 | -0.532827 | 0.42356482 | 0.6216316 | 1.1304034 | 1.1544586 | 0.005297458 |
| 99 | SHROOM4 | -0.5326477 | 0.7133768 | 0.62142235 | 0.9428312 | 1.15407005 | 0.002401062 |
| 100 | PARD3 | -0.53220725 | 0.5818447 | 0.62090844 | 1.0447786 | 1.15311569 | 0.00367403 |
| 101 | FKBP1A | -0.53129697 | 0.777745 | 0.61984646 | 0.8828713 | 1.15114343 | 0.002023111 |
| 102 | ARF5 | -0.53092587 | 0.5830236 | 0.6194135 | 1.0457993 | 1.15033937 | 0.003770117 |
| 103 | MAML3 | -0.5304991 | 0.86048454 | 0.61891556 | 0.7884205 | 1.14941466 | 0.001696417 |

(Table S1 continues on the following page).

**Table S1.** (continued)

| **No.** | **Gene symbol** | **HC mean** | **HC**  **SD** | **ATB mean** | **ATB**  **SD** | **Log_2_**  **(Fold change)** | **Adjusted**  ***p*-value** |
| --- | --- | --- | --- | --- | --- | --- | --- |
| 104 | TAF10 | -0.53012663 | 0.68779826 | 0.61848104 | 0.96885276 | 1.14860767 | 0.002795103 |
| 105 | TICAM2 | -0.5292254 | 0.5383426 | 0.6174297 | 1.0760487 | 1.1466551 | 0.004389443 |
| 106 | BRI3 | -0.5291037 | 0.34677124 | 0.6172877 | 1.1656021 | 1.1463914 | 0.006523178 |
| 107 | PPP1R10 | -0.5289615 | 0.6912706 | 0.61712176 | 0.9676874 | 1.14608326 | 0.002858422 |
| 108 | DHX34 | -0.5275668 | 0.926209 | 0.61549455 | 0.70098656 | 1.14306135 | 0.001604841 |
| 109 | JAG1 | -0.527216 | 0.6741012 | 0.6150854 | 0.9844834 | 1.1423014 | 0.003040292 |
| 110 | TNFAIP2 | -0.52698755 | 0.5751739 | 0.61481875 | 1.0563757 | 1.1418063 | 0.004123666 |
| 111 | LAPTM4A | -0.52696604 | 0.5161558 | 0.6147937 | 1.0918384 | 1.14175974 | 0.004677462 |
| 112 | LOC100507437 | -0.5258737 | 0.72326815 | 0.6135194 | 0.94440967 | 1.1393931 | 0.002781124 |
| 113 | TRIB1 | -0.5257906 | 0.74614877 | 0.6134224 | 0.923264 | 1.139213 | 0.00255973 |
| 114 | PFN1 | -0.52568793 | 0.36869997 | 0.6133026 | 1.1619042 | 1.13899053 | 0.006852813 |
| 115 | LAMTOR5 | -0.5248535 | 0.6389224 | 0.61232907 | 1.0152097 | 1.13718257 | 0.003774947 |
| 116 | KIAA2013 | -0.5248339 | 0.8178165 | 0.6123061 | 0.8501314 | 1.13714 | 0.002124214 |
| 117 | ZFAND2B | -0.5248238 | 0.48018202 | 0.6122943 | 1.1138597 | 1.1371181 | 0.005427157 |
| 118 | CPA3 | -0.5243018 | 0.8132067 | 0.6116854 | 0.85624087 | 1.1359872 | 0.002274075 |
| 119 | PHF23 | -0.5242579 | 0.73013026 | 0.6116342 | 0.94063914 | 1.1358921 | 0.002861155 |
| 120 | CEP55 | -0.522837 | 0.40448356 | 0.6099764 | 1.1513327 | 1.1328134 | 0.006435818 |
| 121 | NCAPH | -0.5221047 | 0.8881124 | 0.60912216 | 0.7673844 | 1.13122686 | 0.002003671 |
| 122 | WAC | -0.52201724 | 0.4347197 | 0.6090201 | 1.1392722 | 1.13103734 | 0.006418189 |
| 123 | PRKAR1A | -0.5219497 | 0.62646097 | 0.6089413 | 1.02843 | 1.130891 | 0.004069187 |
| 124 | GSR | -0.521835 | 0.74748325 | 0.6088076 | 0.92816377 | 1.1306426 | 0.002808028 |
| 125 | GBA | -0.52063584 | 0.6275707 | 0.6074086 | 1.0294664 | 1.12804444 | 0.004178652 |
| 126 | SRA1 | -0.5198768 | 0.7918347 | 0.60652286 | 0.88680875 | 1.12639966 | 0.002625448 |
| 127 | BCKDK | -0.5193572 | 0.70880723 | 0.6059168 | 0.96704 | 1.125274 | 0.003476099 |
| 128 | TMEM179B | -0.51758677 | 0.53049505 | 0.60385126 | 1.0960796 | 1.12143803 | 0.005558364 |
| 129 | ZDHHC12 | -0.5164407 | 0.71379143 | 0.6025141 | 0.9670147 | 1.1189548 | 0.003686204 |

(Table S1 continues on the following page).

| **No.** | **Gene symbol** | **HC mean** | **HC**  **SD** | **ATB mean** | **ATB**  **SD** | **Log_2_**  **(Fold change)** | **Adjusted**  ***p*-value** |
| --- | --- | --- | --- | --- | --- | --- | --- |
| 130 | TUBA4A | -0.51575774 | 0.64268833 | 0.6017174 | 1.025206 | 1.11747514 | 0.004555012 |
| 131 | PPP1R15B | -0.51424366 | 0.5420227 | 0.599951 | 1.093751 | 1.11419466 | 0.005874365 |
| 132 | ERF | -0.5136573 | 0.62037706 | 0.59926695 | 1.0441812 | 1.11292425 | 0.004866104 |
| 133 | FERMT3 | -0.513387 | 0.47001153 | 0.5989515 | 1.1335108 | 1.1123385 | 0.006766866 |
| 134 | ATP6V1F | -0.5133575 | 0.74780387 | 0.598917 | 0.9408091 | 1.1122745 | 0.003588209 |
| 135 | ATP5J2 | -0.51265174 | 0.49513358 | 0.59809375 | 1.1217275 | 1.11074549 | 0.006744052 |
| 136 | FMR1 | -0.5125092 | 0.3798336 | 0.5979274 | 1.1738405 | 1.1104366 | 0.008545354 |
| 137 | TAB3 | -0.51137024 | 0.5757379 | 0.5965986 | 1.0769775 | 1.10796884 | 0.00564852 |
| 138 | MLX | -0.5106458 | 0.7524895 | 0.5957534 | 0.94046324 | 1.1063992 | 0.003617211 |
| 139 | ZRANB2-AS1 | -0.510524 | 0.68101865 | 0.5956113 | 1.0029438 | 1.1061353 | 0.004600027 |
| 140 | LTBR | -0.510111 | 0.7913552 | 0.5951295 | 0.90280896 | 1.1052405 | 0.003317132 |
| 141 | BACH1 | -0.5099349 | 0.70944625 | 0.59492403 | 0.9802241 | 1.10485893 | 0.004273943 |
| 142 | LINC01474 | -0.50970787 | 1.1475503 | 0.59465927 | 6.23E-08 | 1.10436714 | 0.003227 |
| 143 | GAD1 | -0.50949323 | 0.74396783 | 0.5944087 | 0.95014966 | 1.10390193 | 0.003933 |
| 144 | CPO | -0.5093994 | 1.14787 | 0.5942993 | 0 | 1.1036987 | 0.003246 |
| 145 | RBM7 | -0.50910705 | 0.5062413 | 0.5939583 | 1.1203189 | 1.10306535 | 0.007141 |
| 146 | ITM2B | -0.50890565 | 0.43081468 | 0.59372324 | 1.1572423 | 1.10262889 | 0.00812 |
| 147 | RNF13 | -0.50871986 | 0.47401974 | 0.5935065 | 1.1373345 | 1.10222636 | 0.007349 |
| 148 | MSL1 | -0.5084956 | 0.72228587 | 0.59324485 | 0.9711623 | 1.10174045 | 0.004134 |
| 149 | RAB31 | -0.5084737 | 0.4870686 | 0.5932192 | 1.1311051 | 1.1016929 | 0.007319 |
| 150 | RAB21 | -0.5083182 | 0.94664246 | 0.59303784 | 0.707912 | 1.10135604 | 0.002541 |
| 151 | KRT86 | -0.5073761 | 0.32711095 | 0.59193873 | 1.1984935 | 1.09931483 | 0.009527 |
| 152 | ZDHHC18 | -0.50728613 | 0.9458232 | 0.59183383 | 0.7112397 | 1.09911996 | 0.002612 |
| 153 | DUSP6 | -0.5072819 | 0.58428586 | 0.5918289 | 1.0768687 | 1.0991108 | 0.006074 |
| 154 | C10orf131 | -0.5060924 | 0.5921079 | 0.59044117 | 1.0733569 | 1.09653357 | 0.006174 |
| 155 | RNASE2 | -0.5057809 | 0.3587966 | 0.59007776 | 1.1896044 | 1.09585866 | 0.009653 |

**Table S1.** (continued)

(Table S1 continues on the following page).

**Table S1.** (continued)

| **No.** | **Gene symbol** | **HC mean** | **HC**  **SD** | **ATB mean** | **ATB**  **SD** | **Log_2_**  **(Fold change)** | **Adjusted**  ***p*-value** |
| --- | --- | --- | --- | --- | --- | --- | --- |
| 156 | DOK1 | -0.5055972 | 0.66941404 | 0.58986336 | 1.0189316 | 1.09546056 | 0.005164 |
| 157 | NPTN | -0.50556076 | 0.6417747 | 0.5898209 | 1.0397849 | 1.09538166 | 0.005615 |
| 158 | G3BP2 | -0.5044602 | 0.70358837 | 0.5885369 | 0.992943 | 1.0929971 | 0.004839 |
| 159 | CREB5 | -0.5044176 | 0.60519105 | 0.5884872 | 1.0668877 | 1.0929048 | 0.006304 |
| 160 | LOC101928977 | -0.50415117 | 0.8134824 | 0.5881764 | 0.88868797 | 1.09232757 | 0.003687 |
| 161 | STXBP3 | -0.5040615 | 0.53816646 | 0.5880717 | 1.1089661 | 1.0921332 | 0.007142 |
| 162 | NINJ1 | -0.5034591 | 0.876708 | 0.5873689 | 0.81571823 | 1.090828 | 0.003255 |
| 163 | LOC100507006 | -0.50338787 | 0.9114834 | 0.58728594 | 0.76948196 | 1.09067381 | 0.003068 |
| 164 | LLPH | -0.5031436 | 0.5323799 | 0.5870009 | 1.1134076 | 1.0901445 | 0.007296 |
| 165 | SLFN12 | -0.5026969 | 0.6272074 | 0.5864797 | 1.0540218 | 1.0891766 | 0.00603 |
| 166 | PPM1B | -0.50252944 | 0.68976694 | 0.5862844 | 1.0070039 | 1.08881384 | 0.00512 |
| 167 | ASPM | -0.5023021 | 0.74784875 | 0.58601916 | 0.957082 | 1.08832126 | 0.004584 |
| 168 | CDV3 | -0.5019298 | 0.78264815 | 0.58558476 | 0.9241713 | 1.08751456 | 0.004225 |
| 169 | EPOR | -0.50174975 | 0.84274685 | 0.58537465 | 0.85973614 | 1.0871244 | 0.003584 |
| 170 | HJURP | -0.50154936 | 0.5210968 | 0.585141 | 1.1216688 | 1.08669036 | 0.007576 |
| 171 | EMC7 | -0.50116163 | 0.4692306 | 0.58468854 | 1.1488763 | 1.08585017 | 0.008439 |
| 172 | ZNF100 | -0.50112677 | 0.60482526 | 0.58464783 | 1.0713998 | 1.0857746 | 0.006719 |
| 173 | ARF3 | -0.5004588 | 0.6439653 | 0.58386856 | 1.0449775 | 1.08432736 | 0.006203 |
| 174 | FMO2 | -0.50019634 | 0.5722654 | 0.58356243 | 1.0935086 | 1.08375877 | 0.00703 |
| 175 | MIR223 | -0.4997929 | 0.51246846 | 0.5830918 | 1.1285081 | 1.0828847 | 0.008409 |
| 176 | KLHDC8B | -0.49951395 | 0.4077744 | 0.5827663 | 1.1782022 | 1.08228025 | 0.009659 |
| 177 | MORF4L1 | -0.49864718 | 0.4938834 | 0.581755 | 1.1396441 | 1.08040218 | 0.008687 |
| 178 | KIF13A | -0.4984701 | 0.8111516 | 0.58154845 | 0.8999713 | 1.08001855 | 0.004212 |
| 179 | CENPBD1P1 | -0.49820814 | 0.7462704 | 0.5812428 | 0.9644107 | 1.07945094 | 0.005017 |
| 180 | ANKRD1 | -0.49788854 | 0.75087583 | 0.5808699 | 0.9606338 | 1.07875844 | 0.005022 |
| 181 | FAM174A | -0.49771452 | 0.754103 | 0.5806669 | 0.95789105 | 1.07838142 | 0.005021 |

(Table S1 continues on the following page).

| **No.** | **Gene symbol** | **HC mean** | **HC**  **SD** | **ATB mean** | **ATB**  **SD** | **Log_2_**  **(Fold change)** | **Adjusted**  ***p*-value** |
| --- | --- | --- | --- | --- | --- | --- | --- |
| 182 | PRKCD | -0.49737993 | 0.5348021 | 0.58027655 | 1.1191621 | 1.07765648 | 0.008096 |
| 183 | MAPK3 | -0.49731588 | 0.78693646 | 0.5802019 | 0.9267425 | 1.07751778 | 0.004652 |
| 184 | NCF1B | -0.49686626 | 0.60156715 | 0.57967734 | 1.0790125 | 1.0765436 | 0.007308 |
| 185 | CSNK2B | -0.49655572 | 0.8419881 | 0.57931507 | 0.86888176 | 1.07587079 | 0.004048 |
| 186 | DUSP5P1 | -0.4961601 | 0.9358651 | 0.5788535 | 0.7475251 | 1.0750136 | 0.003494 |
| 187 | SNRK | -0.49609095 | 0.9389486 | 0.57877284 | 0.74306846 | 1.07486379 | 0.00348 |
| 188 | ACTA2 | -0.49594513 | 0.50615466 | 0.5786026 | 1.1365283 | 1.07454773 | 0.009037 |
| 189 | ZSWIM6 | -0.49577358 | 0.5854273 | 0.57840246 | 1.0908253 | 1.07417604 | 0.007561 |
| 190 | UBALD2 | -0.49563906 | 0.7722927 | 0.5782456 | 0.94362485 | 1.07388466 | 0.00492 |
| 191 | PTPRE | -0.49537945 | 0.82036597 | 0.5779427 | 0.8947916 | 1.07332215 | 0.004456 |
| 192 | SMPDL3A | -0.4948829 | 0.6843753 | 0.5773635 | 1.021687 | 1.0722464 | 0.006321 |
| 193 | ADAM10 | -0.49442655 | 0.889787 | 0.5768309 | 0.81421536 | 1.07125745 | 0.003942 |
| 194 | HSD3B7 | -0.4942925 | 0.75181115 | 0.5766746 | 0.9648808 | 1.0709671 | 0.005415 |
| 195 | CDC42EP2 | -0.49370047 | 0.74392754 | 0.5759838 | 0.972905 | 1.06968427 | 0.005536 |
| 196 | MSRB1 | -0.49359924 | 0.52954745 | 0.5758657 | 1.1267054 | 1.06946494 | 0.008702 |
| 197 | GFI1B | -0.49325982 | 0.52620727 | 0.5754697 | 1.1289625 | 1.06872952 | 0.008776 |
| 198 | SERINC3 | -0.4929072 | 0.68961215 | 0.5750584 | 1.020158 | 1.0679656 | 0.00654 |
| 199 | NUTF2 | -0.4924992 | 0.7208911 | 0.57458246 | 0.99483156 | 1.06708166 | 0.006087 |
| 200 | RASGRF2-AS1 | -0.49219674 | 0.84776783 | 0.5742296 | 0.8690792 | 1.06642634 | 0.004433 |
| 201 | P2RX1 | -0.4917868 | 0.8539865 | 0.5737513 | 0.8624988 | 1.0655381 | 0.004433 |
| 202 | C19orf38 | -0.4911513 | 0.63857275 | 0.5730099 | 1.0609441 | 1.0641612 | 0.007472 |
| 203 | PPP4R3A | -0.4911364 | 0.6923201 | 0.57299244 | 1.0203454 | 1.06412884 | 0.006755 |
| 204 | SARAF | -0.4905877 | 0.7480472 | 0.57235235 | 0.9735136 | 1.06294005 | 0.00588 |
| 205 | IFNAR1 | -0.49031276 | 0.7311292 | 0.57203156 | 0.9889626 | 1.06234432 | 0.006032 |
| 206 | VAV3-AS1 | -0.48935512 | 0.94842917 | 0.5709142 | 0.7411534 | 1.06026932 | 0.004013 |
| 207 | PELI1 | -0.48901695 | 0.58420825 | 0.57051986 | 1.0999699 | 1.05953681 | 0.00858 |

**Table S1.** (continued)

(Table S1 continues on the following page).

**Table S1.** (continued)

| **No.** | **Gene symbol** | **HC mean** | **HC**  **SD** | **ATB mean** | **ATB**  **SD** | **Log_2_**  **(Fold change)** | **Adjusted**  ***p*-value** |
| --- | --- | --- | --- | --- | --- | --- | --- |
| 208 | KIF5B | -0.48837355 | 0.56960195 | 0.56976914 | 1.1097678 | 1.05814269 | 0.009284 |
| 209 | GAB2 | -0.48834175 | 0.8960364 | 0.569732 | 0.81623983 | 1.05807375 | 0.004489 |
| 210 | GNB1 | -0.48780686 | 0.6383935 | 0.569108 | 1.0653175 | 1.05691486 | 0.007965 |
| 211 | TOX4 | -0.48777232 | 0.67779124 | 0.5690677 | 1.0362005 | 1.05684002 | 0.007324 |
| 212 | PEA15 | -0.48609108 | 0.7805474 | 0.56710625 | 0.94927716 | 1.05319733 | 0.005972 |
| 213 | SULT1A1 | -0.48600775 | 0.5914796 | 0.56700903 | 1.0990548 | 1.05301678 | 0.009014 |
| 214 | PDLIM7 | -0.48434705 | 0.94023126 | 0.5650715 | 0.76226854 | 1.04941855 | 0.004571 |
| 215 | SUOX | -0.48433036 | 0.84298766 | 0.5650521 | 0.88658506 | 1.04938246 | 0.005505 |
| 216 | E2F3 | -0.48398256 | 0.80065405 | 0.5646463 | 0.93230575 | 1.04862886 | 0.006086 |
| 217 | ITGB8 | -0.4838682 | 0.70187515 | 0.5645129 | 1.0222048 | 1.0483811 | 0.00739 |
| 218 | HEXIM1 | -0.4835601 | 0.92511404 | 0.56415343 | 0.78516215 | 1.04771353 | 0.004773 |
| 219 | PPP1R11 | -0.48343834 | 0.74916154 | 0.5640114 | 0.9823237 | 1.04744974 | 0.006804 |
| 220 | WBP1L | -0.4831126 | 0.83467597 | 0.56363136 | 0.89764214 | 1.04674396 | 0.005718 |
| 221 | IL13RA1 | -0.4829068 | 0.84447634 | 0.5633912 | 0.887052 | 1.046298 | 0.005666 |
| 222 | TSG101 | -0.4828389 | 0.88400483 | 0.5633121 | 0.8404137 | 1.046151 | 0.005177 |
| 223 | ARPC2 | -0.48263767 | 0.72709906 | 0.5630773 | 1.0027884 | 1.04571497 | 0.007374 |
| 224 | CASP8 | -0.4820337 | 0.7148726 | 0.5623726 | 1.0139167 | 1.0444063 | 0.00756 |
| 225 | FOS | -0.48201194 | 0.65200365 | 0.5623473 | 1.0628468 | 1.04435924 | 0.008775 |
| 226 | JDP2 | -0.4817176 | 0.7367398 | 0.56200385 | 0.9956671 | 1.04372145 | 0.007144 |
| 227 | MOSPD2 | -0.48142958 | 0.5825209 | 0.56166786 | 1.1102093 | 1.04309744 | 0.00986 |
| 228 | UBAP1 | -0.4812105 | 0.8203557 | 0.5614122 | 0.9158762 | 1.0426227 | 0.006072 |
| 229 | MXD1 | -0.4810879 | 0.6202042 | 0.5612691 | 1.0862359 | 1.042357 | 0.009632 |
| 230 | PPP1R18 | -0.47967097 | 0.89318293 | 0.55961615 | 0.8339224 | 1.03928712 | 0.005476 |
| 231 | CERKL | -0.4794404 | 0.6457225 | 0.55934715 | 1.070558 | 1.03878755 | 0.009255 |
| 232 | C1orf43 | -0.4794371 | 0.6724703 | 0.5593433 | 1.0509204 | 1.0387804 | 0.008642 |
| 233 | CDKN1B | -0.47906098 | 0.8088186 | 0.55890447 | 0.93098587 | 1.03796545 | 0.006451 |

(Table S1 continues on the following page).

**Table S1.** (continued)

| **No.** | **Gene symbol** | **HC mean** | **HC**  **SD** | **ATB mean** | **ATB**  **SD** | **Log_2_**  **(Fold change)** | **Adjusted**  ***p*-value** |
| --- | --- | --- | --- | --- | --- | --- | --- |
| 234 | TMIGD3 | -0.4790546 | 0.83101416 | 0.55889696 | 0.9075995 | 1.03795156 | 0.006267 |
| 235 | STS | -0.478978 | 0.68577856 | 0.5588077 | 1.0412898 | 1.0377857 | 0.008609 |
| 236 | SDCBP | -0.477302 | 0.66561854 | 0.55685234 | 1.0587265 | 1.03415434 | 0.00946 |
| 237 | NEDD9 | -0.4772493 | 0.88941395 | 0.55679077 | 0.84247077 | 1.03404007 | 0.005808 |
| 238 | FAM63B | -0.47640595 | 0.7271332 | 0.555807 | 1.0109433 | 1.03221295 | 0.008332 |
| 239 | ACVR1B | -0.47635385 | 0.7851164 | 0.55574614 | 0.9583923 | 1.03209999 | 0.007266 |
| 240 | MCL1 | -0.4760975 | 0.668413 | 0.55544704 | 1.0581414 | 1.03154454 | 0.009239 |
| 241 | ING1 | -0.47607383 | 0.6443989 | 0.5554195 | 1.0756314 | 1.03149333 | 0.009857 |
| 242 | ZBTB7B | -0.4758989 | 0.75043744 | 0.5552154 | 0.99128395 | 1.0311143 | 0.007904 |
| 243 | CXCL1 | -0.4758936 | 0.7986027 | 0.5552092 | 0.9457708 | 1.0311028 | 0.007217 |
| 244 | ARHGAP25 | -0.47578636 | 0.8473948 | 0.55508405 | 0.8943433 | 1.03087041 | 0.006569 |
| 245 | CHRAC1 | -0.47546718 | 0.80115193 | 0.55471164 | 0.9438122 | 1.03017882 | 0.007257 |
| 246 | SLC25A44 | -0.47391763 | 0.8086048 | 0.5529039 | 0.9384346 | 1.02682153 | 0.007422 |
| 247 | ZMPSTE24 | -0.4738708 | 0.6904523 | 0.55284935 | 1.0440797 | 1.02672015 | 0.009438 |
| 248 | THAP1 | -0.47364673 | 0.73549116 | 0.5525879 | 1.0073668 | 1.02623463 | 0.008712 |
| 249 | LINC01481 | -0.47360644 | 0.7075642 | 0.5525408 | 1.0307865 | 1.02614724 | 0.008969 |
| 250 | HOXA2 | -0.4734438 | 0.7477899 | 0.55235106 | 0.99687445 | 1.02579486 | 0.008321 |
| 251 | GNA15 | -0.47304782 | 0.69427484 | 0.551889 | 1.0421112 | 1.02493682 | 0.00955 |
| 252 | MARCKS | -0.47276476 | 0.746615 | 0.5515588 | 0.9988021 | 1.02432356 | 0.008443 |
| 253 | SUMO1P1 | -0.47256702 | 0.7517654 | 0.5513282 | 0.99448514 | 1.02389522 | 0.008429 |
| 254 | MXD3 | -0.4725435 | 0.80819577 | 0.5513008 | 0.940759 | 1.0238443 | 0.007636 |
| 255 | FNDC3B | -0.47199917 | 0.81484646 | 0.55066574 | 0.9347129 | 1.02266491 | 0.007415 |
| 256 | MALAT1 | -0.4716811 | 0.7716955 | 0.5502946 | 0.97745806 | 1.0219757 | 0.008391 |
| 257 | SLC15A4 | -0.4716389 | 0.7891475 | 0.5502454 | 0.96090657 | 1.0218843 | 0.007955 |
| 258 | UBALD1 | -0.4711186 | 0.9013528 | 0.5496384 | 0.8369726 | 1.020757 | 0.006515 |
| 259 | GTPBP2 | -0.4704791 | 0.7399472 | 0.5488923 | 1.0076036 | 1.0193714 | 0.009216 |

(Table S1 continues on the following page).

**Table S1.** (continued)

| **No.** | **Gene symbol** | **HC mean** | **HC**  **SD** | **ATB mean** | **ATB**  **SD** | **Log_2_**  **(Fold change)** | **Adjusted**  ***p*-value** |
| --- | --- | --- | --- | --- | --- | --- | --- |
| 260 | EZH2 | -0.47011963 | 0.90410876 | 0.5484728 | 0.8350064 | 1.01859243 | 0.006629 |
| 261 | C3orf62 | -0.46975663 | 0.76941586 | 0.54804945 | 0.98212636 | 1.01780608 | 0.008735 |
| 262 | FRMD4B | -0.4694324 | 0.71396685 | 0.5476711 | 1.0308341 | 1.0171035 | 0.009649 |
| 263 | CA8 | -0.46941814 | 0.85040975 | 0.54765457 | 0.90022165 | 1.01707271 | 0.007472 |
| 264 | CSF2RB | -0.46895403 | 0.76419306 | 0.54711306 | 0.98798573 | 1.01606709 | 0.008924 |
| 265 | ALCAM | -0.46852583 | 0.7497552 | 0.5466135 | 1.0015264 | 1.01513933 | 0.009138 |
| 266 | STRIP2 | -0.46835822 | 0.7530605 | 0.5464179 | 0.99880815 | 1.01477612 | 0.009136 |
| 267 | WTAP | -0.4674546 | 0.7946094 | 0.54536366 | 0.9612253 | 1.01281826 | 0.008593 |
| 268 | NKIRAS2 | -0.46729645 | 0.74689746 | 0.5451792 | 1.0056254 | 1.01247565 | 0.009386 |
| 269 | FAM212B | -0.4668544 | 0.93823755 | 0.5446635 | 0.7945836 | 1.0115179 | 0.006753 |
| 270 | ATP6V1A | -0.46634573 | 0.8400855 | 0.54407 | 0.9159441 | 1.01041573 | 0.008063 |
| 271 | PISD | -0.46597496 | 0.83178407 | 0.54363745 | 0.9253698 | 1.00961241 | 0.008209 |
| 272 | ANKRD13D | -0.46537286 | 0.8101012 | 0.542935 | 0.9486459 | 1.00830786 | 0.008794 |
| 273 | CLSPN | -0.46535745 | 0.8480439 | 0.54291695 | 0.9086338 | 1.0082744 | 0.008144 |
| 274 | TFE3 | -0.46501425 | 0.82316357 | 0.54251665 | 0.9357542 | 1.0075309 | 0.008459 |
| 275 | TOPORS | -0.46348095 | 0.88824034 | 0.54072773 | 0.8648351 | 1.00420868 | 0.007796 |
| 276 | ARF1 | -0.46301463 | 0.8483899 | 0.5401837 | 0.9115478 | 1.00319833 | 0.008535 |
| 277 | FAM71D | -0.46280745 | 1.0576698 | 0.53994185 | 0.6000176 | 1.0027493 | 0.006447 |
| 278 | SSH3 | -0.46168852 | 0.8793143 | 0.5386367 | 0.87815624 | 1.00032522 | 0.008188 |
| 279 | ACSS2 | -0.46119887 | 0.84450865 | 0.5380654 | 0.91832024 | 0.99926427 | 0.008895 |
| 280 | PREX1 | -0.4611767 | 0.86736304 | 0.53803945 | 0.89282215 | 0.99921615 | 0.008406 |
| 281 | PAPOLB | -0.46030968 | 1.1952734 | 0.5370279 | 0 | 0.99733758 | 0.008096 |
| 282 | HSH2D | -0.46029574 | 0.8747447 | 0.5370117 | 0.88553494 | 0.99730744 | 0.008476 |
| 283 | C10orf67 | -0.46024248 | 1.1953338 | 0.5369496 | 0 | 0.99719208 | 0.008105 |
| 284 | ARL14 | -0.46011433 | 1.1954489 | 0.5368002 | 6.23E-08 | 0.99691453 | 0.008124 |
| 285 | ZBTB18 | -0.45973596 | 0.9104968 | 0.53635865 | 0.8427107 | 0.99609461 | 0.008168 |

(Table S1 continues on the following page).

**Table S1. (continued)**

| **No.** | **Gene Symbol** | **HC mean** | **HC**  **SD** | **ATB mean** | **ATB**  **SD** | **Log_2_**  **(Fold change)** | **Adjusted**  ***p*-value** |
| --- | --- | --- | --- | --- | --- | --- | --- |
| 286 | P2RY1 | -0.45959288 | 0.8585826 | 0.53619164 | 0.9050208 | 0.99578452 | 0.009025 |
| 287 | ORAI3 | -0.45900345 | 0.9732331 | 0.53550416 | 0.75653934 | 0.99450761 | 0.00756 |
| 288 | CAB39 | -0.45889935 | 0.9604585 | 0.53538257 | 0.77576333 | 0.99428192 | 0.007727 |
| 289 | ABHD4 | -0.45868704 | 0.85127413 | 0.535135 | 0.9143988 | 0.99382204 | 0.009273 |
| 290 | RAB37 | -0.45769334 | 0.83545077 | 0.5339755 | 0.93283355 | 0.99166884 | 0.00964 |
| 291 | MYBL2 | -0.4575421 | 1.0625466 | 0.5337992 | 0.60096925 | 0.9913413 | 0.007157 |
| 292 | C7orf43 | -0.4567905 | 0.8700929 | 0.53292227 | 0.8958956 | 0.98971277 | 0.009154 |
| 293 | EQTN | -0.45610952 | 1.0542431 | 0.5321279 | 0.6209236 | 0.98823742 | 0.007485 |
| 294 | CCDC125 | -0.45330915 | 0.89557725 | 0.5288606 | 0.8707398 | 0.98216975 | 0.009498 |
| 295 | RTCA-AS1 | -0.45183554 | 0.94580823 | 0.52714145 | 0.80780816 | 0.97897699 | 0.009135 |
| 296 | B3GNT8 | -0.44729778 | 0.9744442 | 0.52184737 | 0.773834 | 0.96914515 | 0.009611 |
| 297 | LINC01340 | -0.44534525 | 1.0621808 | 0.51956946 | 0.62645847 | 0.96491471 | 0.009234 |

ATB, active tuberculosis; HC, healthy control; SD, standard deviation.

**Table S2. Diagnostic performance of TB-IEVs as potential biomarkers for active tuberculosis**

| **Markers** | | **EV levels, median (a.u.)** | | | **AUC**  **(95% CI)** | **Cutoff** | **Sensitivity** | **Specificity** | ***p*-value** |
| --- | --- | --- | --- | --- | --- | --- | --- | --- | --- |
|  |  | **HC (n=10)** | **ATB (n=24)** | |  |  |  |  |  |
| **EV**  **Markers** | CD9 | 16.30 | 20.81 | | 0.76 (0.57‒0.95) | 19.15 | 75.0% | 80.0% | 0.016 |
|  | CD63 | 10.00 | 10.34 | | 0.55 (0.33‒0.76) | 10.10 | 54.1% | 50.0% | ns |
|  | CD81 | 23.55 | 24.65 | | 0.51 (0.29‒0.74) | 24.02 | 54.1% | 50.0% | ns |
| **EV markers combination** | | 17.30 | 20.70 | | 0.68 (0.47‒0.90) | 17.25 | 66.7% | 70.0% | ns |
| **TB-IEV**  **markers** | RAB8A | 34.65 | 46.30 | | 0.72 (0.54‒0.89) | 40.94 | 66.7% | 70.0% | 0.0468 |
|  | TYROBP | 27.85 | 87.05 | | 0.96 (0.91‒1.00) | 55.85 | 87.5% | 90.0% | < 0.0001 |
|  | C3AR1 | 50.05 | 84.85 | | 0.95 (0.87‒1.00) | 56.21 | 91.7% | 100.0% | < 0.0001 |
|  | LILRA2 | 9.75 | 33.35 | | 0.83 (0.69‒0.98) | 22.20 | 79.2% | 80.0% | 0.0017 |
| **TB-IEV markers combination** | | 24.90 | | 61.20 | 0.99 (0.96‒1.00) | 33.25 | 95.8% | 90.0% | < 0.0001 |

ns, not significant (p > 0.05). ATB, active tuberculosis; AUC, area under the curve; CI, confidence interval; EV, extracellular vesicle; HC, healthy control; TB-IEV, tubbioerculosis-associated immune cell extracellular vesicle.

**Table S3.** **Clinical diagnoses of patients in the other respiratory disease (ORD) comparator cohort (n = 40)**

| **Patient** | **Clinical diagnosis and concurrent diagnoses or symptoms** |
| --- | --- |
| ORD-1 | Lung carcinoma |
| ORD-2 | Pneumonia |
| ORD-3 | Bronchitis |
| ORD-4 | Bronchiectasis |
| ORD-5 | Bronchitis |
| ORD-6 | Acute bronchitis |
| ORD-7 | Lung carcinoma |
| ORD-8 | Pneumonia |
| ORD-9 | Acute nasopharyngitis |
| ORD-10 | Acute laryngopharyngitis |
| ORD-11 | Pneumonia |
| ORD-12 | Pulmonary embolism, maxillary sinusitis, cough |
| ORD-13 | Pneumonia, bronchitis, pleural effusion, atelectasis, pulmonary embolism |
| ORD-14 | Pneumonia, bronchitis, pulmonary nodule |
| ORD-15 | Pneumonia, bronchitis, sepsis |
| ORD-16 | Pulmonary embolism, sinusitis, cough |
| ORD-17 | Pneumonia, maxillary sinusitis, cough, fever, pulmonary embolism |
| ORD-18 | Bronchitis |
| ORD-19 | Pneumonia, dyspnea |
| ORD-20 | Pneumonia, COPD, asthma |
| ORD-21 | Pneumonia, COPD |
| ORD-22 | COPD, pleural effusion, dyspnea |
| ORD-23 | Idiopathic pulmonary fibrosis, cough, dyspnea |
| ORD-24 | COPD |
| ORD-25 | Pneumonia |
| ORD-26 | Pneumonia, pulmonary edema, respiratory failure, cyanosis, diabetes mellitus |
| ORD-27 | Pneumonia, colitis |
| ORD-28 | Pneumonia, idiopathic pulmonary fibrosis |
| ORD-29 | Bronchitis, pulmonary nodule |
| ORD-30 | Empyema |
| ORD-31 | Pneumonia |
| ORD-32 | Acute interstitial pneumonia |
| ORD-33 | Pneumonia |
| ORD-34 | Pneumonia |
| ORD-35 | Pneumonia |
| ORD-36 | Pneumonia, Sepsis |
| ORD-37 | Acute respiratory distress syndrome |
| ORD-38 | Pneumonia |
| ORD-39 | Acute respiratory distress syndrome |
| ORD-40 | Pneumonia |

ORD, other respiratory disease; COPD, chronic obstructive pulmonary disease; URTI, upper respiratory tract infection. Each patient was classified according to the clinically dominant respiratory diagnosis, defined as the condition principally responsible for the respiratory presentation (outpatient visit or hospital admission) at the time of sampling, as determined by two pulmonologists. Concurrent diagnoses and presenting symptoms are listed for completeness but were not used for classification or counted separately; these include non-respiratory conditions (sepsis in ORD-15 and ORD-36, diabetes mellitus in ORD-26, and colitis in ORD-27). For classification, bronchitis and acute bronchitis were grouped together, and acute nasopharyngitis (ORD-9) and acute laryngopharyngitis (ORD-10) were grouped as URTI. Resulting classification: pneumonia (n = 21), bronchitis (n = 5), COPD (n = 2), acute respiratory distress syndrome (n = 2), lung carcinoma (n = 2), URTI (n = 2), pulmonary embolism (n = 2), idiopathic pulmonary fibrosis (n = 1), acute interstitial pneumonia (n = 1), bronchiectasis (n = 1), and empyema (n = 1).

**Table S4. Diagnostic performance of the TB-IEV multichannel microfluidic chip for discriminating active tuberculosis from other respiratory diseases**

| **Markers** | | **EV levels, median (a.u.)** | | | **AUC**  **(95% CI)** | | | **Cutoff** | **Sensitivity** | | **Specificity** | ***p*-value** |  |
| --- | --- | --- | --- | --- | --- | --- | --- | --- | --- | --- | --- | --- | --- |
|  |  | **ATB (n=25)** | **ORD (n=40)** | |  |  |  |  |  |  |  |  |  |
| **TB marker** | LAM | 63.01 | | 37.93 | | 0.83 (0.73-0.93) | 50.67 | | 76.0% | 70.0% | | < 0.0001 | |
| **TB-IEV**  **markers** | RAB8A | 58.74 | | 41.48 | | 0.93 (0.85-1.00) | 44.78 | | 84.0% | 80.0% | | < 0.0001 | |
|  | TYROBP | 67.19 | | 42.32 | | 0.98 (0.96-1.00) | 49.38 | | 96.0% | 92.5% | | < 0.0001 | |
|  | C3AR1 | 65.29 | | 39.87 | | 0.92 (0.82-1.00) | 49.14 | | 92.0% | 90.0% | | < 0.0001 | |
|  | LILRA2 | 63.35 | | 41.08 | | 0.91 (0.83-1.00) | 50.16 | | 84.0% | 82.5% | | < 0.0001 | |
| **TB-IEV markers combination** | | 60.51 | | 41.34 | | 0.95 (0.88-1.00) | 46.38 | | 92.0% | 97.5% | | < 0.0001 | |

ATB, active tuberculosis; AUC, area under the curve; CI, confidence interval; EV, extracellular vesicle; ORD, other respiratory disease; TB-IEV, tuberculosis-associated immune cell extracellular vesicle.

**Table S5.** **Baseline characteristics of patients with active tuberculosis, healthy controls and patients with other respiratory disease**

| Characteristics | Discovery cohort  (EV surface marker discovery) | |  | Validation cohort  (targeted marker validation) | |  | Application cohort  (commercial ELISA and multichannel chip) | | |
| --- | --- | --- | --- | --- | --- | --- | --- | --- | --- |
|  | ATB (n = 12) | HC (n = 14) |  | ATB (n = 24) | HC (n = 10) |  | ATB (n = 25) | HC (n = 24) | ORD^a^ (n=40) |
| Age (mean ± SD) | 41.0 ± 19.6 | 39.8 ± 10.9 |  | 62.0 ± 21.6 | 22 ± 0.0 |  | 67.0 ± 18 | 49.3 ± 5.3 | 70.2 ± 17.1 |
| Sex (male, %) | 8 (66.7) | 8 (57.1) |  | 16 (66.7) | 2 (20.0) |  | 18 (72.0) | 3 (12.5) | 19 (47.5) |
| MTB PCR positive, n (%) | 12 (100) | N/A |  | 14 (58.3) | N/A |  | 15 (60.0) | N/A | 0 (0) |
| Chest X-ray positive, n (%) | 7 (58.3) | N/A |  | 15 (62.5) | N/A |  | 19 (76.0) | N/A | 0 (0) |
| AFB stain positive, n (%) | 2 (16.7) | N/A |  | 4 (16.7) | N/A |  | 5 (20.0) | N/A | 0 (0) |
| AFB agar culture positive, n (%) | 7 (58.3) | N/A |  | 6 (25.0) | N/A |  | 8 (32.0) | N/A | 0(0) |
| AFB broth culture positive, n (%) | 10 (83.3) | N/A |  | 18 (75.0) | N/A |  | 14 (56.0) | N/A | 0 (0) |

ATB, active tuberculosis; HC, healthy control; ORD, other respiratory disease; MTB, *Mycobacterium tuberculosis*; AFB, acid-fast bacilli; SD, standard deviation; N/A, not applicable; ND, not determined. The discovery, validation, and application cohorts comprised independent participants with no overlap. ᵃActive TB was excluded in all ORD patients on clinical, microbiological, and radiological grounds and by clinical course; none received anti-tuberculosis treatment during follow-up.

**Table S6.** **Conventional diagnostic findings and diagnostic basis in patients with active tuberculosis (n = 25)**

| **Patient** | **MTB PCR** | **Chest X-ray** | **AFB staining** | **AFB agar culture** | **AFB broth culture** | **Diagnostic basis** |
| --- | --- | --- | --- | --- | --- | --- |
| ATB-1 | Negative | Positive | Negative | Negative | Negative | Clinically diagnosed |
| ATB-2 | Negative | Positive | Negative | Negative | Negative | Clinically diagnosed |
| ATB-3 | Positive | Negative | Negative | Negative | Positive | Bacteriologically confirmed  (PCR, culture) |
| ATB-4 | Positive | Negative | Negative | Negative | Negative | Bacteriologically confirmed (PCR) |
| ATB-5 | Positive | Negative | Positive | Negative | Positive | Bacteriologically confirmed  (PCR, culture) |
| ATB-6 | Positive | Positive | Negative | Negative | Positive | Bacteriologically confirmed  (PCR, culture) |
| ATB-7 | Negative | Positive | Negative | Negative | Negative | Clinically diagnosed |
| ATB-8 | Positive | Positive | Negative | Negative | Negative | Bacteriologically confirmed (PCR) |
| ATB-9 | Negative | Positive | Negative | Negative | Negative | Clinically diagnosed |
| ATB-10 | Negative | Positive | Positive | Positive | Positive | Bacteriologically confirmed (Culture) |
| ATB-11 | Negative | Positive | Negative | Negative | Positive | Bacteriologically confirmed (Culture) |
| ATB-12 | Positive | Positive | Negative | Negative | Positive | Bacteriologically confirmed  (PCR, culture) |
| ATB-13 | Negative | Positive | Negative | Negative | Negative | Clinically diagnosed |
| ATB-14 | Negative | Positive | Negative | Negative | Negative | Clinically diagnosed |
| ATB-15 | Positive | Positive | Negative | Negative | Negative | Bacteriologically confirmed (PCR) |
| ATB-16 | Positive | Negative | Positive | Positive | Positive | Bacteriologically confirmed  (PCR, culture) |
| ATB-17 | Positive | Negative | Negative | Negative | Negative | Bacteriologically confirmed (PCR) |
| ATB-18 | Positive | Negative | Negative | Positive | Positive | Bacteriologically confirmed  (PCR, culture) |
| ATB-19 | Positive | Positive | Positive | Positive | Positive | Bacteriologically confirmed  (PCR, culture) |
| ATB-20 | Positive | Positive | Negative | Positive | Positive | Bacteriologically confirmed  (PCR, culture) |
| ATB-21 | Negative | Positive | Negative | Negative | Negative | Clinically diagnosed |
| ATB-22 | Positive | Positive | Positive | Positive | Positive | Bacteriologically confirmed  (PCR, culture) |
| ATB-23 | Positive | Positive | Negative | Positive | Positive | Bacteriologically confirmed  (PCR, culture) |
| ATB-24 | Negative | Positive | Negative | Negative | Positive | Bacteriologically confirmed (Culture) |
| ATB-25 | Positive | Positive | Negative | Positive | Positive | Bacteriologically confirmed  (PCR, culture) |

ATB, active tuberculosis; MTB, *Mycobacterium tuberculosis*; AFB, acid-fast bacilli; PCR, polymerase chain reaction. The diagnostic basis was assigned independently of the TB-IEV assay, in accordance with World Health Organization criteria. Patients with a positive MTB culture (agar or broth) were classified as bacteriologically confirmed (culture); patients with a positive MTB PCR result but negative cultures were classified as bacteriologically confirmed (PCR); and patients without bacteriological confirmation who showed clinical and radiological findings compatible with active tuberculosis together with a favorable response to anti-tuberculosis treatment were classified as clinically diagnosed. Overall, 18 of 25 patients (72.0%) had bacteriologically confirmed ATB and 7 (28.0%) were clinically diagnosed.

**Table S7.** **Antibodies against EV and TB-IEV markers**

| Target | Vendor | Origin | Ab Type | Concentration |
| --- | --- | --- | --- | --- |
| CD9 | BD (555370) | Mouse | IgG1 | 0.5 mg/mL |
| CD63 | BD (556019) | Mouse | IgG1 | 0.5 mg/mL |
| CD81 | BD (555675) | Mouse | IgG1 | 0.5 mg/mL |
| RAB8A | Invitrogen (MA5-31883) | Mouse | IgG2 | 1.0 mg/mL |
| TYROBP | Invitrogen (PA5-26861) | Rabbit | IgG | 0.5 mg/mL |
| C3AR1 | Abcam (ab103629) | Rabbit | IgG1 | 1.0 mg/mL |
| LILRA2 | Sigma-Aldrich (SAB4500198) | Rabbit | IgG | 1.0 mg/mL |

EV, extracellular vesicle; HC, healthy control; TB-IEV, tuberculosis-associated immune cell extracellular vesicle

**Table S8. List of primary antibodies used for western blotting and fluorescence analyses**

| Target | Vendor | Origin | Dilution ratio |
| --- | --- | --- | --- |
| CD63 | BD (557305) | Mouse | 1:500 |
| CD81 | Invitrogen (MA5-13548) | Mouse | 1:500 |
| β-actin | Cell Signaling (58169) | Mouse | 1:1000 |
| RAB8A | Cell Signaling (6975S) | Rabbit | 1:1000 |
| TYROBP | Invitrogen (PA5-26861) | Rabbit | 1:1000 |
| C3AR1 | Abcam (ab103629) | Rabbit | 1:1000 |
| LILRA2 | Sigma-Aldrich (SAB4500198) | Rabbit | 1:1000 |
| CD63-PE-Cy7 | BD (561982) | Rabbit | 1:100 |
| CD9-Percp | BD (561329) | Mouse | 1:100 |

**Table S9. List of secondary antibodies used for western blotting and fluorescence analyses**

| Target | Vendor | Dilution ratio |
| --- | --- | --- |
| Rabbit IgG-FITC | BD (554020) | 1:200 |
| Mouse IgG-FITC | BD (555988) | 1:200 |
| Rabbit IgG-HRP | Invitrogen (31460) | 1:10000 |
| Mouse IgG-HRP | Cell Signaling (7076S) | 1:3000 |

**Table S10.** **List of ELISA kits**

| Target | ELISA kits | Vendor |
| --- | --- | --- |
| RAB8A | Human RAB8A (Ras-related protein Rab-8A) ELISA Kit | FineTest (EH1615) |
| TYROBP | Human TYROBP (TYRO protein tyrosine kinase  -binding protein) ELISA Kit | FineTest (EH1444) |
| C3AR1 | Human C3AR1 (C3a anaphylatoxin chemotactic receptor) ELISA Kit | FineTest (EH14227) |
| LILRA2 | Human LILRA2(Leukocyte immunoglobulin-like receptor subfamily A member 2) ELISA Kit | FineTest (EH2451) |
| LAM | Human Lipoarabinomannan (LAM) ELISA Kit | AFG Scientific (EK711140) |

**Figure S1. Heatmap of differentially expressed mRNA in whole blood samples of healthy controls (n = 14) and patients with active tuberculosis (n = 12) (fold change 1.5, *p* < 0.01 and** **false discovery rate [FDR] < 0.05).**

**Figure S2. Characterization of plasma-derived extracellular vesicles from patients with active tuberculosis.** (A) Nanoparticle tracking analysis of total EVs isolated from ATB plasma, showing a size distribution peak around 100–150 nm and the corresponding particle concentration. The inset displays individual particle intensities plotted against size. (B) Transmission electron microscopy images confirming the typical spherical morphology of EVs (arrows). Scale bar: 200 nm. ATB, active tuberculosis; EVs, extracellular vesicles.

**Figure S3. Immunoprecipitation with magnetic beads for TB-IEVs isolation.** (A) Representative flow cytometric analysis of beads coated with EV (CD9, CD63, and CD81) and TB-IEV markers (RAB8A, TYROBP, C3AR1, and LILRA2). (B) Bar plots showing fluorescence intensity for EV-capture bead populations (mean ± SD). (C) Analysis of time-dependent stability in immunoprecipitation assays. MFI, median fluorescence intensity; n.s., not significant; EVs, extracellular vesicles; SD, standard deviation; TB-IEVs, tuberculosis-associated immune cell extracellular vesicles.

**Figure S4. Head-to-head comparison of reference tests and EV marker performance for ATB diagnosis.** Positivity of TB-IEV markers (RAB8A, TYROBP, C3AR1, and LILRA2), conventional EV markers (CD9, CD63, and CD81), and clinical diagnostic data (MTB PCR, AFB staining, culture, and X-ray results). ATB, active tuberculosis; TB-IEV, tuberculosis-associated immune cell extracellular vesicles.

**Figure S5. Computational fluid dynamic simulation of the multichannel chip.** (A) Velocity contours illustrating uniform flow distribution across all channels. (B) Streamline and flow vector plots demonstrating smooth trajectories and absence of stagnation zones, validating the design for efficient parallel operation. Simulations were performed at a flow rate of 1 mL/min.

**Figure S6. Magnetic field distribution and capture performance of magnetic beads in the multichannel chip.** (A) 3D Schematic of the multichannel chip integrated with an array of magnets, with the cross-sectional analysis line indicated (a–a′). (B) Simulated magnetic flux density distribution along the chip, demonstrating a uniform magnetic field across parallel channels. (C) Magnetic flux density profile along the cross-sectional line (a–a′), showing localized peaks corresponding to each channel. (D) Capture efficiency of the magnetic beads at different concentrations and incubation times.

**Figure S7. Uncropped original blots for western blot analysis related to the Figure 2E**

**Figure S8. Uncropped original blots for western blot analysis related to the Figure 3D**
